# Human RSPO1 Mutation Represses Beige Adipocyte Thermogenesis and Contributes to Diet‐Induced Adiposity

**DOI:** 10.1002/advs.202207152

**Published:** 2023-02-08

**Authors:** Yingkai Sun, Juan Zhang, Jie Hong, Zhongyun Zhang, Peng Lu, Aibo Gao, Mengshan Ni, Zhiyin Zhang, Huanjie Yang, Juan Shen, Jieli Lu, Wenzhi Xue, Qianqian Lv, Yufang Bi, Yi Arial Zeng, Weiqiong Gu, Guang Ning, Weiqing Wang, Ruixin Liu, Jiqiu Wang

**Affiliations:** ^1^ Department of Endocrine and Metabolic Diseases Shanghai Institute of Endocrine and Metabolic Diseases Ruijin Hospital Shanghai Jiao Tong University School of Medicine 197 Ruijin 2nd Road Shanghai 200025 P. R. China; ^2^ Shanghai National Clinical Research Center for Metabolic Diseases Key Laboratory for Endocrine and Metabolic Diseases of the National Health Commission of the PR China Shanghai National Center for Translational Medicine Shanghai 200025 P. R. China; ^3^ BGI Genomics BGI‐Shenzhen Shenzhen 860755 P. R. China; ^4^ State Key Laboratory of Cell Biology CAS Center for Excellence in Molecular Cell Science Institute of Biochemistry and Cell Biology Chinese Academy of Sciences University of Chinese Academy of Sciences Shanghai 200031 P. R. China

**Keywords:** obesity, pathogenic gene, RSPO1, thermogenesis, whole‐exome sequencing, Wnt signaling

## Abstract

Recent genetic evidence has linked WNT downstream mutations to fat distribution. However, the roles of WNTs in human obesity remain unclear. Here, the authors screen all Wnt‐related paracrine factors in 1994 obese cases and 2161 controls using whole‐exome sequencing (WES) and identify that 12 obese patients harbor the same mutations in RSPO1 (p.R219W/Q) predisposing to human obesity. RSPO1 is predominantly expressed in visceral fat, primarily in the fibroblast cluster, and is increased with adiposity. Mice overexpressing human *RSPO1* in adipose tissues develop obesity under a high‐fat diet (HFD) due to reduced brown/beige fat thermogenesis. In contrast, *Rspo1* ablation resists HFD‐induced adiposity by increasing thermogenesis. Mechanistically, RSPO1 overexpression or administration significantly inhibits adipocyte mitochondrial respiration and thermogenesis via LGR4–Wnt/*β*‐catenin signaling pathway. Importantly, humanized knockin mice carrying the hotspot mutation (p.R219W) display suppressed thermogenesis and recapitulate the adiposity feature of obese carriers. The mutation disrupts RSPO1's electrostatic interaction with the extracellular matrix, leading to excessive RSPO1 release that activates LGR4–Wnt/*β*‐catenin signaling and attenuates thermogenic capacity in differentiated beige adipocytes. Therefore, these findings identify that gain‐of‐function mutations and excessive expression of RSPO1, acting as a paracrine Wnt activator, suppress fat thermogenesis and contribute to obesity in humans.

## Graphical abstract

RSPO1 p.R219W mutation, first identified in severely obese young patients, disrupts its electrostatic interaction with the extracellular matrix,resulting in long‐distance diffusion and more arrivals of RSPO1 protein to LGR4‐positive preadipocytes, and consequently enhances its inhibition on mitochondria biogenesis and thermogenesis during browning. This is referred to as a new paradigm in obesity development due to a paracrine disorder.

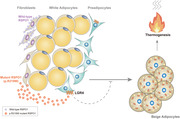

## 1 Introduction

Obesity occurs when excess energy is stored in white adipose tissues (WAT), while thermogenic adipocytes in brown adipose tissues (BAT) promote energy expenditure to reduce adiposity. Under exogenous and physiological stimuli, such as coldness, feeding and exercise, inducible browning (beige) adipocytes that play similar roles as classical brown adipocytes are sporadically raised within human WAT.^[^ ^1^ ^]^ Beige adipocytes with enriched mitochondria profoundly dissipate energy into heat and have been considered a promising and viable approach for treating obesity, which has fueled the field over the past decade.^[^ ^2^ ^]^ Whereas, the browning capacity of WAT as well as BAT activity is significantly suppressed in subjects with obesity,^[^ ^3^ ^]^ which is a well‐described clinical characteristic but lacks a defined etiology.^[^ ^4^ ^]^ A number of mammalian genetic models ranging from mouse, rat, and even pig have demonstrated that artificially manipulated gene mutations can lead to obesity by inhibiting the thermogenesis of brown/beige adipocytes.^[^ ^5^ ^]^ However, essential evidence of pathogenic mutations in human genes that control thermogenic capacity at the organism level is still insufficient, raising uncertainty about the clinical significance and potential applications of targeting beige/brown fat in the diagnosis and treatment of obesity.^[^ ^6^ ^]^

Adipose tissue is a highly plastic organ consisting of mixed populations of cell lineages and extracellular matrix (ECM), functioning together to maintain metabolic homeostasis.^[^ ^7^ ^]^ Several studies on the intrinsic mechanisms within adipocytes have elucidated a huge molecular network that regulates the browning program.^[^ ^8^ ^]^ Recently, emerging evidence has identified coordinated paracrine factors among (pre)adipocytes themselves,^[^ ^9^ ^]^ their surrounding cells (such as fibroblasts and immune cells),^[^ ^7b^ ^]^ and the ECM components (such as laminin *α*4).^[^ ^10^, ^11^ ^]^ External cues and drugs can change the paracrine homeostasis of adipocyte microenvironmental niches in WAT, altering their thermogenic capacity.^[^ ^12^ ^]^ For example, mirabegron, which acts as a *β*3‐adrenergic receptor (*β*3‐AR) agonist and has been intensively studied in humans and mice, effectively promotes the browning program of subcutaneous WAT along with ECM remodeling.^[^ ^13^ ^]^

Paracrine factors, physically tethered by ECM components to act over a restricted short distance,^[^ ^14^ ^]^ can regulate white‐to‐brown fate determination and cell differentiation of fat progenitors.^[^ ^7a^ ^]^ The collapse of their concentration gradients would lead to a paracrine disorder that damages the patterning and growth of organisms.^[^ ^14^, ^15^ ^]^ Previous biological and genetic association evidence prioritizes one family of paracrine factors—WNTs together with their modulators—involved in adipogenesis, fat distribution and the development of obesity,^[^ ^16^ ^]^ whereas the feasibility of directly demonstrating the causality between WNTs mutation and obesity at the organismal level remains elusive. One challenge is the lack of deep‐sequencing data in a large‐scale population enriched with genetic factors, such as an early‐onset or extremely obese cohort.^[^ ^17^ ^]^ Novel genetic evidence can provide critical information for clinical physicians and basic researchers to understand whether pathogenic mutations in certain paracrine factors in WNT signaling could lead to human obesity by regulating energy homeostasis. To this end, we performed whole‐exome sequencing (WES) in a large sample‐size cohort of young, severely obese subjects and lean controls^[^ ^16^, ^18^ ^]^ targeting WNT‐related genes and identified a gain‐of‐function mutation (p.R219W) in the human *RSPO1* gene that specifically disrupts its electrostatic interaction with the ECM and leads to increased RSPO1 release to the extracellular space, amplifying WNT signaling of the targeted preadipocytes and inhibiting the thermogenic capacity of beige adipocytes, and consequently contributes to diet‐induced obesity. These findings indicate that RSPO1 may represent a novel pathogenic mutation to diagnose obesity and a therapeutic target to intervene in obesity.

## 2 Results

### 2.1 Gain‐of‐Function RSPO1 Mutations Are Identified in Young Obesity

**Figure 1:**
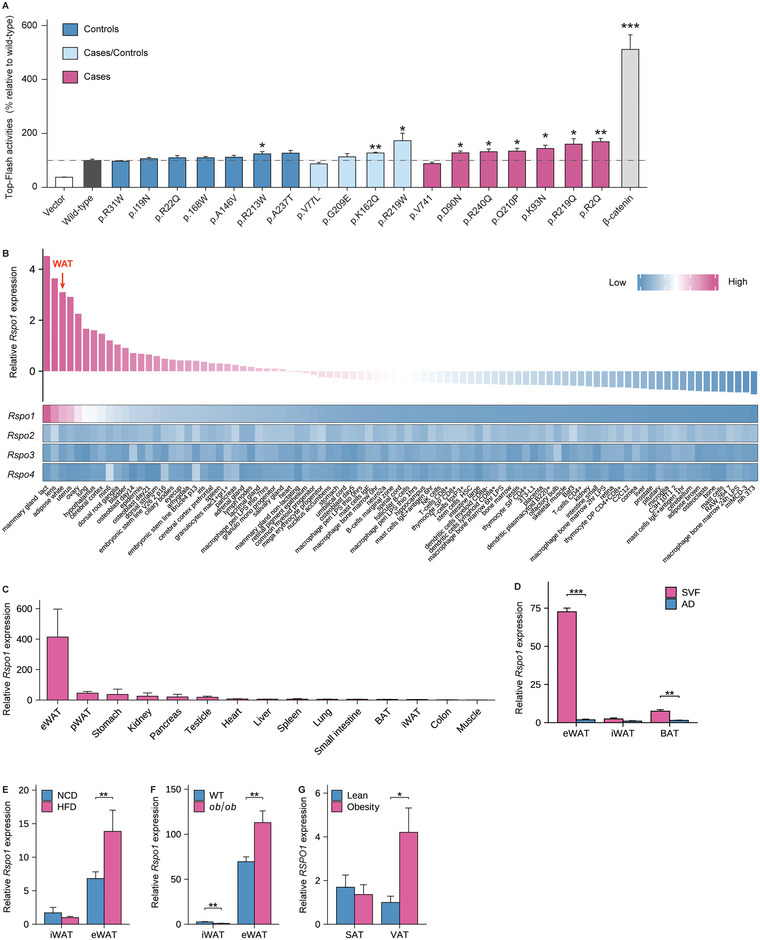
RSPO1 is enriched in visceral fat and increases with adiposity. A) TOP‐Flash luciferase reporter assay of HEK293T cells transfected with plasmids of wild‐type RSPO1 and 18 rare/low‐frequency RSPO1 nonsynonymous variants described in Table 1, respectively. *β*‐catenin plasmids were applied as positive control (*n* = 5 per group). Statistical significances were calculated between wild‐type and each variant using unpaired Student's *t*‐test. B) Heat map representing the expression of mouse Rspo1/2/3/4 in diverse tissues and cell lines, as analyzed using a publicly available microarray data set (GSE10246). C) Quantitative PCR validation of Rspo1 expression in multiple tissues of 8‐week‐old male C57BL/6J mice (*n* = 3–6 per group). D) Rspo1 mRNA expression in the stromal vascular fractions (SVF) and mature adipocytes (AD) of eWAT, iWAT, and BAT, respectively (*n* = 3 per group). E,F) Rspo1 mRNA expression in iWAT and eWAT of mice fed normal chow diet (NCD) and high‐fat diet (HFD) (*n* = 13–16 per group) (E), or in that of wild‐type (WT) and *ob*/*ob* mice (*n* = 10 per group) (F). G) RSPO1 mRNA expression in the subcutaneous adipose tissue (SAT) and visceral adipose tissues (VAT) of obese subjects versus normal weight controls (*n* = 10–17 per group). eWAT, epididymal white adipose tissue; pWAT, pararenal white adipose tissue; iWAT, inguinal white adipose tissue; BAT, brown adipose tissue. SAT, subcutaneous adipose tissue; VAT, visceral adipose tissue. Data are shown as the mean ± sem. *p* values were calculated using unpaired Student's *t*‐test. *p < 0.05; **p < 0.01; ***p < 0.001.

To investigate the potential involvement of genetic mutations of WNT‐related factors in human obesity, the coding regions of 19 WNTs, 6 WNT activators (including RSPOs, etc.) and 16 WNT inactivators (including SFRPs, etc.) (Table S1, Supporting Information) were systemically analyzed in our in‐house whole‐exome sequencing (WES) cohort of 1944 young, severely obese cases (mean ± sem: BMI, 35.43 ± 0.11 kg m^−2^; age, 24.91 ± 0.18 years) and 2161 healthy lean controls (BMI, 21.04 ± 0.03 kg m^−2^; age, 42.80 ± 0.24 years), the sequencing information of which has been described in our previous studies.^[^ ^16^, ^18^ ^]^ We included all of the rare/low‐frequency variations with minor allele frequency (MAF) < 5% in controls, including missense, indel and frameshift, into the association analysis and identified four missense mutations in *RSPO4*, *SFRP5*, *WNT10A* and *RSPO1* genes that were associated with a changed risk of obesity (*p* < 0.05). Among them, RSPO1 p.R219W mutation exhibited the highest odds ratio (OR), and the association remained significant after adjustment for sex (Table S2, Supporting Information). In addition, RSPO4 p.L54H and SFRP5 p.P232Q mutations were enriched in controls, and the WNT10A p.R171C mutation has been repeatedly reported to highly predispose to non‐syndromic tooth agenesis but not adiposity.^[^ ^19^ ^]^ Therefore, RSPO1 p.R219W together with p.R219Q mutations identified at the same site were further investigated (**Table** **1**). These two p.R219 residue mutants (p.R219W/Q) were significantly associated with increased obesity risk (crude OR = 4.47; 95% confidential interval [CI], 1.20–24.70; P = 0.017) (Table 1), and they remained significant after adjustment (adjusted OR = 3.09; 95% CI, 2.07–4.01; P = 0.014) (Figure S1, Supporting Information). These results were further validated as compared with the general Chinese population^[^ ^20^ ^]^ (OR = 4.65; 95% CI, 2.02–10.34; P = 1.63 × 10^−4^) (Table 1) and East Asians from the gnomAD database (OR = 20.21; 95% CI, 5.45–111.99; P = 1.18 × 10^−7^) (Table S3, Supporting Information), respectively. We next evaluated the functional changes of all the 18 nonsynonymous mutations in RSPO1 identified in our cohort (Table 1) using the TOP‐Flash reporter system. p.R219W/Q and six other missense mutations presented in cases exhibited increased activating effects on WNT signaling than wild‐type RSPO1. In comparison, six of seven missense mutations that only occurred in lean carriers did not result in activity changes (**Figure** **1**A). Among them, p.R219W/Q mutants showed a relatively higher activation (Figure 1A). Thus, gain‐of‐function mutations in the *RSPO1* gene confer a risk for human obesity.

**Table 1:** Rare and low‐frequency RSPO1 nonsynonymous variants identified in young Chinese with obesity and controls

Position^a)^	Nucleotide change^b)^	AA change^b)^	Cases (*n* = 1944)	Lean controls (*n* = 2161)	ChinaMAP controls (*n* = 10 588)	Case _freq	Lean Control _freq	ChinaMAP controls _freq	*p* value^c)^	OR (95% CI)^c)^	*p* value^d)^	OR (95% CI)^d)^
1:38095329	C > T	R2Q	2	1	2	0.0005	0.0002	0.0001	0.61	2.22 (0.12, 131.20)	0.12	5.45 (0.40, 75.09)
1:38095278	A > T	I19N	0	2	1	0	0.0005	0.0000	0.50	0.00 (0.00, 5.92)	1.00	0.00 (0.00, 211.71)
1:38095269	C > T	R22Q	0	1	1	0	0.0002	0.0000	1.00	0.00 (0.00, 43.33)	1.00	0.00 (0.00, 211.71)
1:38095243	G > A	R31W	0	2	4	0	0.0005	0.0002	0.50	0.00 (0.00, 5.92)	1.00	0.00 (0.00, 8.25)
1:38082222	C > T	V74I	1	0	1	0.0003	0	0.0000	0.47	Inf. (0.03, Inf.)	0.29	5.45 (0.07, 426.22)
1:38082212	G > A	P77L	1	1	7	0.0003	0.0002	0.0003	1.00	1.11 (0.01, 87.25)	1.00	0.78 (0.02, 6.06)
1:38082174	C > T	D90N	1	0	0	0.0003	0	0	0.47	Inf. (0.03, Inf.)	0.16	Inf. (0.14, Inf.)
1:38082163	C > A	K93N	1	0	0	0.0003	0	0	0.47	Inf. (0.03, Inf.)	0.16	Inf. (0.14, Inf.)
1:38079564	G > A	A146V	0	2	1	0	0.0005	0.0000	0.50	0.00 (0.00, 5.92)	1.00	0.00 (0.00, 211.71)
1:38079517	T > G	K162Q	115	111	649	0.0298	0.0261	0.0306	0.30	1.16 (0.88, 1.53)	0.76	0.96 (0.78, 1.18)
1:38079496	G > A	R169W	0	1	1	0	0.0002	0.0000	1.00	0.00 (0.00, 43.33)	1.00	0.00 (0.00, 211.71)
1:38078593	C > T	G209E	2	3	10	0.0005	0.0007	0.0005	1.00	0.74 (0.06, 6.48)	1.00	1.09 (0.12, 5.12)
1:38078590	T > G	Q210P	1	0	0	0.0003	0	0	0.47	Inf. (0.03, Inf.)	0.16	Inf. (0.14, Inf.)
1:38078582	T > A	R213W	0	1	0	0	0.0002	0	1.00	Inf. (0.00, 43.33)	‐	‐
1:38078564	G > A	R219W	11	3	13	0.0028	0.0007	0.0006	0.03	4.09 (1.08, 22.88)	4.26 × 10^−4^	4.63 (1.87, 11.22)
1:38078563	C > T	R219Q	1	0	1	0.0003	0	0.0000	0.47	Inf. (0.03, Inf.)	0.29	0.29 (0.07, 426.22)
1:38078510	C > T	A237T	0	2	3	0	0.0005	0.0001	0.50	0.00 (0.00, 5.92)	1.00	0.00 (0.00, 13.18)
1:38078500	C > T	R240Q	1	0	0	0.0003	0	0	0.47	Inf. (0.03, Inf.)	0.16	Inf. (0.14, Inf.)

^a)^ NCBI Build 37.

^b)^ Variations are based on RefSeq records NM_001038633.4 and NP_001033722.1, respectively. Freq, allele frequency.

^c)^ Cases versus lean controls.

^d)^ Cases versus ChinaMAP controls. The data of East Asian and all gnomAD samples are from gnomAD v2.1.1 (https://gnomad.broadinstitute.org/gene/). Inf., infinity.

### 2.2 RSPO1 Is Enriched in Visceral Fat and Increased During Adiposity

RSPO1 is a secretory protein that plays determinant roles in intestinal organoid growth^[^ ^21^ ^]^ and female sex determination.^[^ ^22^ ^]^ To further examine the biological function of RSPO1 in obesity, we first analyzed *RSPO1* expression using online microarray data across various organs and cell lines.^[^ ^23^ ^]^ We found that *RSPO1*, but not other *RSPO* members (*RSPO*2–4), was specifically and highly expressed in WAT (Figure 1B). This was confirmed by qPCR analysis in tissues of 8‐week‐old male C57BL/6J mice, among which epididymal WAT (eWAT) displayed the highest expression level of endogenous *Rspo1* (Figure 1C). We also compared the *Rspo1* expression between mature adipocyte and stromal vascular fractions (SVFs) isolated from various adipose depots and found that *Rspo1* in SVFs contributed to the dominant part (≈90%) of its total abundance in eWAT depot (Figure 1D). Further analysis with murine single‐cell sequencing data^[^ ^24^ ^]^ revealed that *Rspo1*‐positive cells were preferentially enriched in two clusters of eWAT‐derived SVFs, with high abundance in fibroblasts (FBs) and relatively low expression in adipose stem cells (ASCs) (Figure S2A–D, Supporting Information). Consistently, *Rspo1* was also more highly expressed in the SVFs of visceral fat than subcutaneous fat in humans (Figure S2E, Supporting Information).^[^ ^25^ ^]^

We subsequently examined *Rspo1* expression in the WATs of two obese models, high‐fat diet (HFD)‐induced mice and *L* *eptin*‐deficient obese (*ob*/*ob*) mice, and found that *Rspo1* was significantly increased in the eWATs of both models (Figure 1E,F). Importantly, obese subjects also displayed higher *RSPO1* expression in the visceral WAT (vWAT) than lean controls (Figure 1G). However, we did not observe any difference in circulating concentrations of RSPO1 between young subjects with overweight/obesity and age‐ and sex‐matched lean controls, despite the fact it could be detectable in plasma at a low level (pg mL^−1^) (Table S4, Supporting Information). These results indicate that *RSPO1* is highly expressed in visceral fat and dominantly produced by SVF cells (especially fibroblasts), and its local but not circulating levels are positively associated with the development of obesity.

### 2.3 Human RSPO1 Overexpression Promotes HFD‐Induced Obesity and Suppresses Brown/Beige Fat Thermogenesis

**Figure 2:**
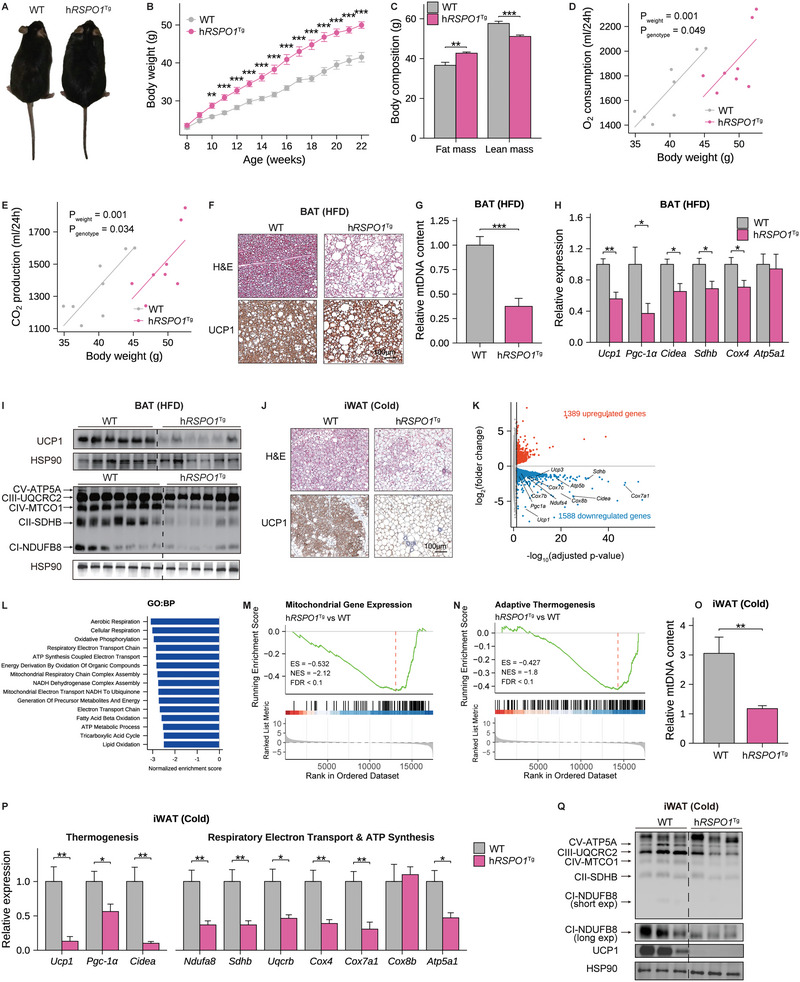
Human *RSPO1* overexpression promotes diet‐induced obesity by suppressing adipose thermogenesis. A–C) A representative global image (A), body weight curve (B), and body composition (C) of human *RSPO1* transgenic (h*RSPO1* ^Tg^) and WT littermate mice fed HFD for 14 weeks (*n* = 8 per group). D,E) ANCOVA analysis of 24‐hour O_2_ consumption (D) and CO_2_ production (E) with body weight as a covariate in HFD‐fed WT and h*RSPO1* ^Tg^ mice (*n* = 8 per group). F) Representative images of H&E staining and UCP1 immunohistochemical staining of BAT in HFD‐fed WT and h*RSPO1* ^Tg^ mice (*n* = 4 per group). Scale bar, 100 µm. G) The mitochondrial DNA (mtDNA) content of BAT in HFD‐fed WT and h*RSPO1* ^Tg^ mice (*n* = 6–7 for per group). H,I) Quantitative PCR analysis (H) and Western blotting (I) of mitochondrial respiratory complexes and thermogenic genes in BAT of HFD‐fed WT and h*RSPO1* ^Tg^ mice (*n* = 6–7 per group). J) Representative images of H&E staining and UCP1 immunohistochemical staining in iWAT of WT and h*RSPO1* ^Tg^ mice exposed to chronic cold stimulation (4 °C) for 10 days (*n* = 4 per group). Scale bar, 100 µm. K) The volcano plot of genes differentially expressed in iWAT of h*RSPO1* ^Tg^ versus WT mice under chronic cold stimulation (*n* = 3 per group). Biomarkers associated with mitochondrial functions were indicated. L) The top‐downregulated (FDR < 0.05) pathways revealed by GSEA based on GO:BP database in iWAT of h*RSPO1* ^Tg^ versus WT mice under chronic cold stimulation. (M) and (N) GSEA results of mitochondrial gene expression (M) and adaptive thermogenesis (N) in iWAT of h*RSPO1* ^Tg^ versus WT mice under chronic cold stimulation. O–Q) Relative mtDNA content (O), quantitative PCR analysis (P), and Western blotting analysis (Q) of mitochondrial respiratory complexes and thermogenic genes in iWAT of h*RSPO1* ^Tg^ and WT mice under chronic cold stimulation (*n* = 7–8 per group). Data are shown as the mean ± sem, and statistical differences between genotypes were assessed by unpaired Student's *t*‐test (B,C,G,H,O,P); FDR below 0.05 was considered as the criteria for evaluating differentially expressed genes between genotypes (K); FDR below 0.1 was considered as statistical significance in GSEA (L–N). *p < 0.05; **p < 0.01; ***p < 0.001.

To investigate whether increased *RSPO1* in adipose tissue can induce obesity, we generated an *aP2* promoter‐driven human *RSPO1* (h*RSPO1*) transgenic mouse model (h*RSPO1* ^Tg^) in which h*RSPO1* was effectively overexpressed in three adipose tissues (Figure S3A,B, Supporting Information). We found that h*RSPO1* ^Tg^ mice exhibited comparable body weights to wild‐type mice fed normal chow diet (Figure S3C, Supporting Information) but gained more body weight when challenged with HFD (**Figure** **2**A,B), with increased fat mass content and decreased lean mass content (Figure 2C). The weight of inguinal WAT (iWAT) and BAT was also significantly increased in h*RSPO1* ^Tg^ mice (Figure S3D, Supporting Information). Next, we analyzed the energy balance differences between two genotype mice fed HFD. While no significant alterations of cumulative food intake, fecal calorie loss and physical activities were detected in h*RSPO1* ^Tg^ mice (Figure S3E–G, Supporting Information), ANCOVA analysis of the indirect calorimetric measurements showed a lower O_2_ consumption and CO_2_ production in h*RSPO1* ^Tg^ mice after adjustment for body weight (Figure 2D,E). To further exclude the potential confounding effect of body weight changes on energy expenditure, we repeated indirect calorimetric measurements on HFD‐fed wild‐type and h*RSPO1* ^Tg^ mice prior to their body weight divergence. These mice were pre‐stimulated with CL316243, a selective *β*3‐adrenergic receptor agonist, to boost their thermogenic capacities. Consistently, h*RSPO1* ^Tg^ mice exhibited decreased O_2_ consumption, CO_2_ production, and energy expenditure than wild‐type mice (Figure S3H–J, Supporting Information). These results suggested that human *RSPO1* overexpression in adipose tissues is sufficient to reduce energy expenditure and promote HFD‐induced obesity.

We next examined BAT activity and the browning capacity of WAT, which are crucial for regulating energy expenditure and obesity.^[^ ^6b^ ^]^ We found that brown adipocytes in h*RSPO1* ^Tg^ mice exhibited a remarkably enlarged cell size, expanded lipid droplets, as well as decreased levels of UCP1 protein, a marker of brown/beige adipocytes, when compared with wild‐type mice (Figure 2F). The gene expression profile of BAT further revealed that multiple genes and pathways related to mitochondrial respiratory chain complex assembly, mitochondrial gene expression, and thermogenesis were significantly downregulated when *RSPO1* was overexpressed (Figure S3K,L, Supporting Information). These results were then validated by the reduced mitochondria content and attenuated mRNA and protein levels of the mitochondrial respiratory chain, such as complex I–V proteins: NDUFB8, SDHB, UQCRC2, MTCO1 and ATP5A, and thermogenic genes (Figure 2G–I). Similar results were obtained under cold challenge (Figure S4A, Supporting Information). These findings suggested that RSPO1 may play inhibitory roles in mitochondrial respiration and thermogenic activities of brown adipocytes.

To further evaluate the effects of RSPO1 overexpression on the thermogenic capacity of browning/beige adipocytes, we next compared the histological changes and gene expression profile of the iWAT from both genotypes under cold challenge. The cold‐induced browning changes, including reduced adipocyte volumes, increased small multilocular lipid droplets and enhanced UCP1 protein levels, were remarkably suppressed in iWAT of h*RSPO1* ^Tg^ mice (Figure 2J). The RNA sequencing data further revealed that the expression of 2,977 genes was significantly changed, including 1,588 downregulated and 1,389 upregulated genes, in iWAT of h*RSPO1* ^Tg^ mice (Figure 2K and Figure S4B, Supporting Information). Gene set enrichment analysis (GSEA) indicated that the downregulated pathways were most related to mitochondrial activities, including cellular respiration, oxidative phosphorylation, mitochondrial respiratory chain complex assembly, as well as substrates oxidation (Figure 2L and Figure S4C, Supporting Information). In further, the gene sets that represent mitochondrial gene expression and adaptive thermogenesis were also inhibited in h*RSPO1* ^Tg^ mice (Figure 2M,N). These findings were further validated by mRNA quantification and protein measurements of the genes involved in thermogenesis and mitochondrial respiration, including *Ucp1*, *Pgc‐1α*, *Cidea* and complex I–V genes (Figure 2P,Q). Consistently, we also observed that the mitochondrial DNA contents were significantly reduced in iWAT of h*RSPO1* ^Tg^ mice under cold exposure (Figure 2O). With respect to eWAT, similar results were observed but with relatively slight differences in thermogenic gene expression between the two genotypes (Figure S4D–G, Supporting Information), which is possibly attributed to the pre‐existing high‐level endogenous Rspo1 expression in eWAT (Figure 1B,C). In addition, when stimulated with CL316243, suppressed thermogenic changes were consistently observed in iWAT of h*RSPO1* ^Tg^ mice compared to wild‐type mice, including enlarged adipocytes and reduced thermogenic gene expression (Figure S4H–M, Supporting Information). Taken together, these results suggested that excess RSPO1 expression in adipose tissue reduces mitochondrial respiration and thermogenic capacity of brown/beige adipocytes.

### 2.4 Rspo1 Ablation Promotes Adipose Thermogenesis and Resists HFD‐Induced Obesity

**Figure 3:**
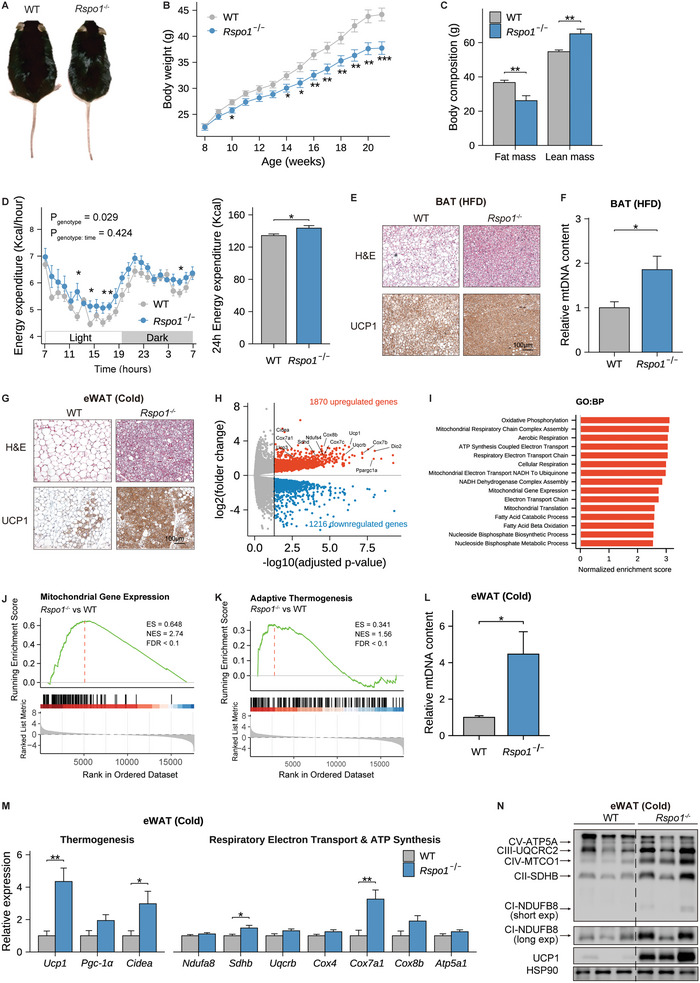
*Rspo1* deficiency promotes adipose thermogenesis and resists HFD‐induced obesity. A–C) A representative global image (A), body weight curve (*n* = 15–22 per group) (B), and body composition (*n* = 7–8 per group) (C) of *Rspo1* ^−/−^ and WT mice fed HFD for 13 weeks. D) Hourly (left) and total (right) energy expenditure over 24 h between *Rspo1* ^−/−^ and WT mice fed with 1‐week HFD (*n* = 8 per group). The hourly measurements were assessed by two‐way ANOVA model to evaluate the interaction between genotype and time, and pairwised *t*‐test with Benjamini–Hochberg correction was used as post‐hoc test for evaluating the differences between genotypes in each hour. The total energy expenditure over 24 h was compared with unpaired Student's *t*‐test. E) Representative images of H&E staining and UCP1 immunohistochemical staining in BAT of WT and *Rspo1* ^−/−^ mice fed HFD (*n* = 4 per group). Scale bar, 100 µm. F) The mtDNA content of BAT in *Rspo1* ^−/−^ and WT mice fed HFD (*n* = 7–8 per group). G) Representative images of H&E staining and UCP1 immunohistochemical staining in eWAT of WT and *Rspo1* ^−/−^ mice exposed to chronic cold stimulation (4 °C) for 10 days (*n* = 4 per group). Scale bar, 100 µm. H) The volcano plot of genes differentially expressed in eWAT of *Rspo1* ^−/−^ versus WT mice under chronic cold stimulation (*n* = 3 per group). Biomarkers associated with mitochondrial functions were indicated. I) The top‐upregulated pathways (FDR < 0.05) revealed by GSEA analysis based on GO:BP database in eWAT of *Rspo1* ^−/−^ versus WT mice under chronic cold stimulation. J,K) GSEA results of mitochondrial gene expression (J) and adaptive thermogenesis (K) in eWAT of *Rspo1* ^−/−^ versus WT mice under chronic cold stimulation. L,N) Relative mtDNA content (L), quantitative PCR analysis (M), and Western blotting (N) analysis of mitochondrial respiratory complexes and thermogenic genes in eWAT of *Rspo1* ^−/−^ and WT mice under chronic cold stimulation (*n* = 7–8 per group). Data are shown as the mean ± sem, and statistical differences between genotypes were assessed by unpaired Student's *t*‐test (B,C,F,L,M); FDR below 0.05 was considered as the criteria for evaluating differential expressed genes between genotypes (H); FDR below 0.1 was considered as statistical significance in GSEA (I–K). *p < 0.05; **p < 0.01; ***p < 0.001.

To further investigate the potential anti‐obesity effect of blocking endogenous *Rspo1*, we generated *Rspo1* knockout (*Rspo1* ^−/−^) mice (Figure S5A, Supporting Information) and confirmed that endogenous *Rspo1* was effectively deleted in *Rspo1* ^−/−^ mice (Figure S5B, Supporting Information). Similar to h*RSPO1* ^Tg^ mice, *Rspo1* ^−/−^ mice exhibited comparable body weight to their wild‐type littermates under normal chow diet (Figure S5C, Supporting Information); however, they displayed a reduced body weight under HFD feeding (**Figure** **3**A,B). The whole‐body fat mass and the weight of iWAT and eWAT pads were also significantly reduced in *Rspo1* ^−/−^ mice (Figure 3C and Figure S5D, Supporting Information). Further, prior to the divergence of body weight between two genotypes of mice fed HFD, the energy expenditure appeared to be higher in *Rspo1* ^−/−^ mice compared to controls (Figure 3D and Figure S5E,F, Supporting Information), while no significant changes of cumulative food intake, fecal energy loss or physical activities were observed (Figure S5G–I, Supporting Information). These results indicate that the ablation of endogenous *Rspo1* increases energy expenditure and resists HFD‐induced adiposity.

Consistently, the brown fat of *Rspo1* ^−/−^ mice exhibited more dense droplets, higher UCP1 protein levels, and increased mitochondrial abundance than wild‐type mice (Figure 3E,F). To further evaluate the effects of *Rspo1* deficiency on the thermogenic capacity, we challenged mice with coldness and CL316243 stimulation, respectively. Under acute cold stress, *Rspo1* ^−/−^ mice maintained a higher rectal temperature, indicating an enhanced cold tolerance (Figure S6A, Supporting Information). Moreover, when exposed to long‐term coldness, *Rspo1* ^−/−^ mice exhibited a strongly browning program in eWAT, exhibiting much smaller and condensed adipocytes with robustly increased UCP1 protein levels (Figure 3G). The RNA sequencing data further showed that the expression of 3,086 genes was significantly changed, including 1,870 upregulated and 1,216 downregulated genes, in eWAT of *Rspo1* ^−/−^ mice as compared with wild‐type mice (Figure 3H and Figure S6B, Supporting Information). GSEA analysis of differential genes indicated that most of the upregulated pathways were associated with mitochondrial respiration as well as adaptative thermogenesis in *Rspo1* ^−/−^ mice (Figure 3I and Figure S6C, Supporting Information). Consistently, the gene sets representing mitochondrial gene expression and adaptive thermogenesis were also significantly enhanced in *Rspo1* ^−/−^ mice (Figure 3J,K). These changes were also confirmed by mitochondrial DNA measurement, mRNA quantification, and protein measurements (Figure 3L–N). Similarly, when compared to the changes in eWAT, the cold‐induced browning changes were less profound in iWAT of *Rspo1* ^−/−^ mice (Figure S6D–G, Supporting Information), possibly due to a low endogenous Rspo1 expression in iWAT (Figure 1B,C). We further found that *β*3‐AR activation also led to a more pronounced increase in mitochondrial and thermogenic gene expression in eWAT (Figure S6H–J, Supporting Information) relative to iWAT (Figure S6K–M, Supporting Information) of *Rspo1* ^−/‐^ mice. These data suggest that endogenous *Rspo1* ablation promotes white‐to‐brown fat conversion, especially in visceral fat, contributing to enhanced thermogenesis and resistance to HFD‐induced obesity.

### 2.5 Human RSPO1 Protein Suppresses Beige Adipocyte Thermogenesis via LGR4–Wnt/*β*‐Catenin Signaling

**Figure 4:**
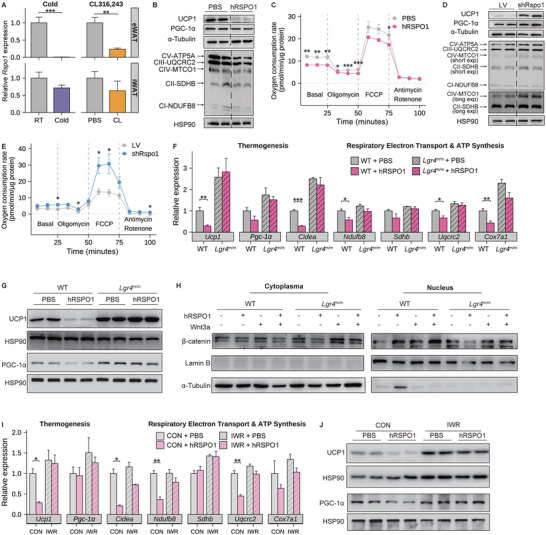
Human RSPO1 protein suppresses beige adipocyte thermogenesis in an Lgr4/*β*‐catenin dependent manner. A) Quantitative PCR analysis of Rspo1 mRNA levels in eWAT (upper panels) and iWAT (bottom panels) in response to either chronic cold exposure (right panels) (*n* = 7–8 per group) or *β*3‐AR agonist (CL316243) injection (right panels) (*n* = 6 per group). B,C) Changes of mitochondrial respiratory complexes and thermogenic proteins (UCP1 and PGC‐1*α*) (B) and OCR (C) in fully differentiated beige adipocytes with human RSPO1 (hRSPO1) recombinant protein or PBS treatment (*n* = 5 per group). D,E) Changes of mitochondrial respiratory complexes and thermogenic proteins (D) and OCR (E) in fully differentiated beige adipocytes treated with Rspo1‐shRNA (shRspo1) or vector‐shRNA (LV) (*n* = 4 per group). F,G) Quantitative PCR analysis of thermogenic genes and mitochondrial respiratory complexes (F) and protein quantification of UCP1 and PGC‐1*α* (G) in fully differentiated beige adipocytes derived from WT and *Lgr4* ^m/m^ mice in response to hRSPO1 or PBS treatment, respectively (*n* = 4–6 per group). H) Alterations of cytoplasmic and nuclear *β*‐catenin protein in the SVFs derived from WT and *Lgr4* ^m/m^ mice in response to hRSPO1, Wnt3a, and their combinations. I,J) Quantitative PCR analysis of thermogenic genes and mitochondrial respiratory complexes (I) and protein quantification of UCP1 and PGC‐1*α* (J) in fully differentiated beige adipocytes treated with hRSPO1 or PBS in the presence or absence of IWR‐endo1, respectively (*n* = 3 per group). WT, wild‐type mice; *Lgr4* ^m/m^, *Lgr4* mutant mice. Data are shown as the mean ± sem. Statistical differences between groups were assessed by unpaired Student's *t*‐test (A,C,E,F,I). *p < 0.05; **p < 0.01; ***p < 0.001.

To evaluate the response of endogenous *Rspo1* to browning cues, we examined *Rspo1* mRNA expression in eWAT and iWAT under coldness or CL316243 stimulation. As a result, *Rspo1* mRNA levels were decreased in eWAT but not significantly changed in iWAT during cold‐ or CL316243‐induced browning adipocyte activation (**Figure** **4**A). We subsequently performed in vitro studies to explore the biological roles of human RSPO1 (hRSPO1) in the browning process. SVF cells containing preadipocytes from iWAT were isolated and incubated with a brown adipocyte differentiation cocktail in the presence or absence of hRSPO1 recombinant protein (Figure S7A, Supporting Information). In line with the in vivo experiments, the fully differentiated beige adipocytes treated with hRSPO1 exhibited a reduction in mRNA and protein levels of thermogenic and mitochondrial genes, including UCP1, PGC‐1*α* and multiple mitochondrial respiratory chain proteins in complex I–V (Figure 4B and Figure S7B, Supporting Information). Meanwhile, no apparent changes were observed in Oil red O staining of full‐differentiated beige adipocytes (Figure S7C, Supporting Information). We next performed cellular respiratory measurements in beige adipocytes with or without hRSPO1 treatment. We found that hRSPO1‐treated cells displayed a significantly lower oxygen consumption rate (OCR) at both basal and uncoupled respiration stages and a decreased trend of maximal respiration (Figure 4C). In contrast, when the endogenous *Rspo1* was silenced by a specific *Rspo1*‐shRNA lentivirus (Figure S7D, Supporting Information), the mRNA and protein expression of mitochondrial and thermogenic genes in beige adipocytes were increased (Figure 4D and Figure S7E, Supporting Information). Consistently, the OCRs of uncoupled and maximal cellular respiration were also increased in beige adipocytes with *Rspo1* deficiency (Figure 4E). Together with the in vivo data, these results suggest that hRSPO1 recombinant protein suppresses mitochondrial and thermogenic capacities of beige adipocytes, and the ablation of endogenous *Rspo1* can enhance the browning program, reinforcing its role as a novel regulator in mitochondrial respiration, including coupling and uncoupling process, in beige adipocytes.

Theoretically, the biological action of RSPO1 can be mediated by several membrane proteins, such as LGR4/5/6 and ZNRF3/RNF43 E3 ligases, that amplify the activation of Wnt signaling.^[^ ^21^ ^]^ We next evaluated the expression pattern of these membrane candidates in SVF fractions and mature adipocytes of eWAT and found that LGR4 protein was more highly and specifically expressed in SVFs than the other molecules (Figure S7G, Supporting Information). Of note, *Lgr4*‐positive cells were primarily localized in the two clusters of eWAT‐derived SVFs (Figure S7H,I, Supporting Information), which are classified as proliferating and differentiating ASCs (Pro. & Diff. ASC) and are highly induced by CL316243.^[^ ^24^ ^]^ Despite with low expression of the ligand (*Rspo1*) (Figure S7B, Supporting Information), iWAT‐derived SVFs displayed relatively high expression of the receptor (*Lgr4*) (Figure S7H, Supporting Information). We next added hRSPO1 protein to the iWAT‐derived SVFs of wild‐type (*Lgr4* ^+/+^) and *Lgr4*‐deficient (*Lgr4* ^m/m^) mice. In the presence of the *Lgr4* receptor, hRSPO1 consistently inhibited the mRNA expression levels of thermogenic and mitochondrial genes, including *Ucp1, Cidea* and multiple mitochondrial respiratory chain components (*Ndufb8* and *Uqcrc2*), while this inhibitory effect was abolished in the absence of *Lgr4* receptor (Figure 4F). In addition, hRSPO1 suppressed the protein levels of UCP1 and PGC‐1*α* protein in a similar LGR4‐dependent manner (Figure 4G). The key regulators of adipocyte differentiation, such as FAPB4 and PPAR*γ*, did not show significant changes with hRSPO1 treatment (Figure S7J, Supporting Information). These results suggest that the inhibitory effect of RSPO1 on mitochondria gene expression and the thermogenesis of beige adipocytes largely depends on the *Lgr4* receptor.

Translocation of *β*‐catenin from the cytoplasm into the nucleus is the key step of canonical Wnt signaling activation.^[^ ^21^ ^]^ To determine whether *β*‐catenin was further involved in the inhibitory effects of RSPO1/LGR4 on beige adipocytes, we assessed nuclear *β*‐catenin protein levels in response to treatment with hRSPO1 in iWAT SVFs from wild‐type and *Lgr4* ^m/m^ mice, respectively. In the presence of Lgr4, both hRSPO1 and its combination with Wnt3a induced more accumulation of nuclear *β*‐catenin, whereas this activating effect was diminished in the absence of *Lgr4* (Figure 4H). Furthermore, we added IWR‐endo1, a canonical Wnt/*β*‐catenin pathway inhibitor,^[^ ^26^ ^]^ to SVF cells to assess whether RSPO1's effect was also dependent on *β*‐catenin activation. In accordance with *Lgr4* ablation, the chemical blockade of *β*‐catenin activity also attenuated the inhibitory effects of RSPO1 on the expression of the thermogenic and mitochondrial genes in beige adipocytes (Figure 4I,J). Taken together, these data indicate that RSPO1 suppresses the mitochondrial and thermogenic capacity of beige adipocytes via the LGR4‐canonical Wnt/*β*‐catenin signaling pathway.

### 2.6 p.R219W/Q Mutants Increase RSPO1 Protein Secretion by Disrupting their Binding with the ECM

**Figure 5:**
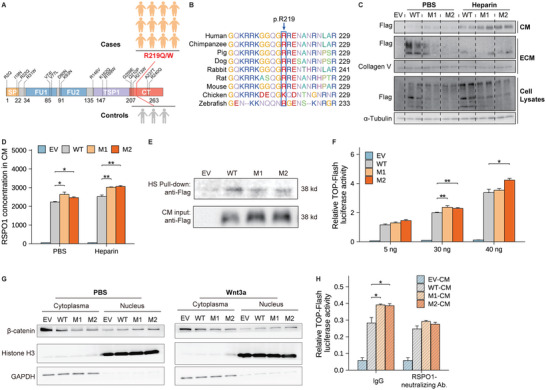
RSPO1 p.R219W/Q mutations disrupt their electrostatic interaction with the ECM and activate Wnt pathway. A) Schematic representation of the full‐length human RSPO1 protein and the location of p.R219W/Q mutations in the C‐terminal (CT) region with a cluster of positively charged amino acid residues (Figure S8, Supporting Information). SP, N‐terminal signal peptide; FR, furin‐like domains; TSR, thrombospondin protein 1 domain. 12 obese cases and 3 lean controls harboring the mutations were identified in GOCY cohort (Figure S1, Supporting Information). B) The consensus sequence of the conserved Arginine 219 (R219) residue in RSPO1 protein across different species. C) Protein secretion of wild‐type RSPO1 (WT) and the two mutants (M1, p.R219W; M2, p.R219Q) in conditioned medium (CM) and by extracellular matrix (ECM) and cell lysates in the absence or presence of 50 µg mL^−1^ heparin, respectively. EV, empty vector. D) RSPO1 protein levels in the conditioned media of the indicated groups (Figure 5C) measured by ELISA (*n* = 3 per group). E) Heparan sulfate (HS) pull‐down assay (Figure S8C, Supporting Information) of RSPO1–HS complex obtained from co‐incubation of HS‐beads and conditioned media (CM) derived from HEK293T cells transfected with wild‐type or mutant RSPO1 plasmids, respectively. Both CM (as Input) and HS‐beads put‐down (as Pull‐down) fractions were immunoblotted with the anti‐Flag antibody. F) A TOP‐Flash luciferase reporter assay was performed in HEK293T cells. The wild‐type and the two mutant RSPO1 plasmids were used for the transfection at the indicated dosages (5, 30, and 40 ng of expression plasmids), and pRL‐SV40 (expressing Renilla luciferase) was used as a normalized control (*n* = 3 per group). A representative result of three independent experiments is shown. G) The *β*‐catenin translocation examination was performed in HEK293T cells transfected with wild‐type or the two mutant RSPO1 plasmids, and treated with PBS or 100 ng mL^−1^ Wnt3a, respectively. H) A TOP‐Flash luciferase reporter assay was performed in HEK293T cells treated with conditioned media, collected from HEK293T cells transfected with empty vector, wild‐type and two mutant RSPO1 plasmids, in the presence or absence of 2 µg mL^−1^ RSPO1 neutralizing antibody (*n* = 3–4 per group). Data are shown as the mean ± sem. Statistical differences between groups were assessed by unpaired Student's *t*‐test (D,F,H) *p < 0.05; **p < 0.01; ***p < 0.001.

Based on the in vivo and in vitro experimental results, we established RSPO1 as an essential suppressor of fat thermogenesis whose overexpression promotes HFD‐induced obesity. To further establish the causality of the hotspot mutation p.R219W/Q in RSPO1 identified in obese subjects (**Figure** **5**A) in thermogenesis inhibition, we next explored the functional changes of p.R219W/Q mutants. Of note, the R219 residue is located in the C‐terminal region of RSPO1 (Figure 5A) and is conserved across various species but less conserved among RSPO1–4 members (Figure 5B and Figure S8A,B, Supporting Information). The C‐terminal region of RSPO1 contains a cluster of basic amino acid residues (positively charged arginine [R] and lysine [K]) (Figure S8A, Supporting Information), forming a region with a high density of positive charges that facilitates their electrostatic binding to heparan sulfate proteoglycans (HSPGs),^[^ ^27^ ^]^ thereby restricting RSPO1 local diffusion within the ECM. Either tryptophan (W) or glutamine (Q) substitution is considered to eliminate the positive charge characterization of the R219 site. When we transfected the two RSPO1 mutants and wild‐type plasmids into human embryonic kidney 293T (HEK293T) cells, we found that the mutations led to more RSPO1 protein release into the conditioned media together with less reservation in cell lysates and ECM than wild‐type RSPO1 (Figure 5C). These effects became more profound when the cells were treated with heparin, which possesses a high density of negative charges and can competitively disrupt the electrostatic interaction between positively charged secreted proteins and the ECM.^[^ ^28^ ^]^ A higher level of RSPO1 protein was detected in the conditioned media of two RSPO1 mutants than the wild‐type controls (Figure 5C). These findings are in line with the previous report in which the addition of soluble heparin can significantly enhance the release of RSPO1, RSPO2, and RSPO3 proteins into the conditioned media.^[^ ^29^ ^]^ Consistent results were also observed with the ELISA kit specific for human RSPO1 proteins (Figure 5D), further confirming the increased release of RSPO1 proteins from their secreted cells transfected with the two mutants. More importantly, we validated the direct interaction between RSPO1 protein and heparin sulfate (HS) components of HSPG by incubating HS beads with the cell lysates of HEK293T cells that were transfected with flagged wild‐type RSPO1 (Figure S8C,D, Supporting Information). To further evaluate the binding ability change of RSPO1 p.R219W/Q mutants to HS components, we collected the conditioned media from HEK293T cells transfected with flagged wild‐type or two mutant RSPO1 plasmids and incubated them with HS. As a result, the HS‐bound RSPO1 mutants were reduced compared with wild‐type RSPO1 (Figure 5E).

In further, overexpression of the two RSPO1 mutants enhanced *β*‐catenin/TCF‐mediated transcriptional activity (Figure 5F), which was additionally validated by increased *β*‐catenin accumulation in the nucleus driven by the mutants (Figure 5G). Similarly, conditioned media collected from p.R219W/Q mutant‐transfected cells also increased TOP‐Flash reporter transcriptional activity, while this effect was abolished upon treatment with anti‐RSPO1 neutralizing antibody (Figure 5H).^[^ ^30^ ^]^ Taken together, these results suggest that p.R219W/Q mutations identified in human obesity disrupt their affinity to HSPG and thus increase RSPO1 protein release from ECM to extracellular space, consequently over‐activating Wnt/*β*‐catenin signaling.

### 2.7 Homologous Rspo1 p.R219W Mutation Leads to Obesity In Vivo

**Figure 6:**
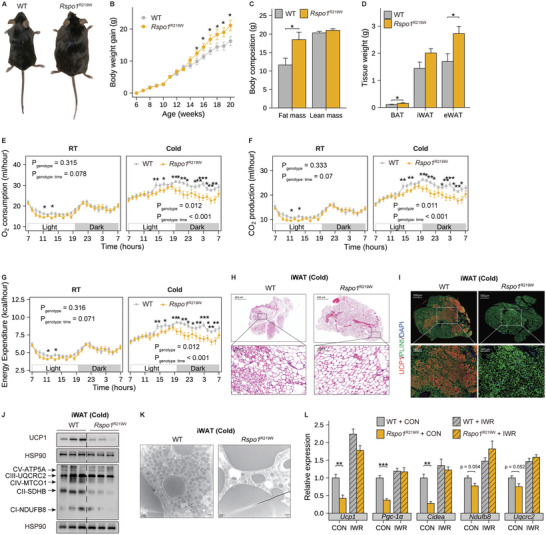
Homologous Rspo1 p.R219W mutation suppresses fat thermogenesis and leads to obesity in vivo. A–D) A representative global image (A), body weight curve (*n* = 9–11 per group) (B), body composition (C), and the weight of three fat tissues (D) of female homozygous *Rspo1* ^R219W^ and WT littermate mice fed HFD for 10 weeks (*n* = 8 per group). E–G) O_2_ consumption, CO_2_ production, and energy expenditure of WT and *Rspo1* ^R219W^ mice fed chow diet shifting from room temperature (22 °C, RT) to cold conditions (4 °C, cold) (*n* = 12 per group). The hourly measurements were assessed by two‐way ANOVA model to evaluate the interaction between genotype and time, and pairwised *t*‐test with Benjamini–Hochberg correction was used as post‐hoc test to evaluate the differences between genotypes in each hour. H–K) Representative images of H&E staining (H), UCP1 immunofluorescence staining (I), protein expression changes of mitochondrial respiratory complexes and thermogenic genes (J), and electron microscopic images of mitochondria (K) in iWAT of WT and *Rspo1* ^R219W^ mice under chronic cold stimulation (4 °C) for 10 days (*n* = 3 per group). UCP1 (red) and perilipin protein (green) were used to indicate beige adipocytes and lipid droplets, respectively. Scale bars are indicated in the panels. L) Quantitative PCR analysis of thermogenic genes in fully differentiated beige adipocytes derived from the iWAT of WT and *Rspo1* ^R219W^ mice in the presence or absence of IWR‐endo1 treatment (*n* = 4 per group). Data are shown as the mean ± sem, and statistical significances between groups were assessed by unpaired Student's *t*‐test (B–D,L). *p < 0.05; **p < 0.01; ***p <0.001.

To further establish the effects of RSPO1 p.R219W mutation on obesity in vivo, we thus produced a p.R219W point mutation knock‐in mouse model using the CRISPR–Cas9 approach, to recapitulate the homologous mutation that occurs in humans faithfully. Similar to the phenotypes in h*RSPO1* ^Tg^ mice, the homozygous p.R219W mutation knock‐in (*Rspo1* ^R219W^) mice exhibited no difference in body weight from their wild‐type littermates under normal chow diet, except for slightly lower body weight in male *Rspo1* ^R219W^ mice in the early stage of life (Figure S9A, Supporting Information); however, under HFD condition, female *Rspo1* ^R219W^ mice gained more body weight (**Figure** **6**A,B and Figure S9B, Supporting Information), with a higher fat mass as well as higher weight of eWAT and BAT (Figure 6C,D), as compared to their wild‐type littermates.

Next, we observed a significant reduction of O_2_ consumption, CO_2_ production, and energy expenditure in *Rspo1* ^R219W^ mice under acute cold exposure but no significant change under room temperature (Figure 6E–G). The rectal temperature was also lower in *Rspo1* ^R219W^ mice in response to acute cold challenge (Figure S9C, Supporting Information). No significant differences in cumulative food intake, fecal calorie excretion, or physical activity were observed between the two genotypes (Figure S9D–F, Supporting Information). In consistence, *Rspo1* ^R219W^ mice exhibited an attenuated browning program in WATs, featured with larger unilocular adipocytes (Figure 6H), weaker staining of the browning marker protein (UCP1) (Figure 6I and Figure S9G, Supporting Information), decreased multiple mitochondrial respiratory chain components in complex I–V (Figure 6J), and reduced mitochondrial content (Figure 6K) as compared to those of wild‐type littermates. Similar changes were also observed in *Rspo1* ^R219W^ mice under CL316243 stimulus (Figure S9H–K, Supporting Information). These findings indicate that the p.R219W mutation identified in the human *RSPO1* gene gains an enhanced capacity to suppress the thermogenesis and mitochondria content of beige fat and accelerates obesity development.

As we only observed increased adiposity in female *Rspo1* ^R219W^ mice but not in males, we next examined the prevalence of p.R219W/Q mutation in females and males in our cohort and did not observe a sex‐specific prevalence of these mutations (0.42% in females versus 0.27% in males, P = 0.60). We speculated that the sex dimorphism in the obesogenic effect of p.R219W in mice might be due to the more robust thermogenic capacity of the beige/BAT in female than male rodents,^[^ ^31^ ^]^ which may amplify the suppressive effects of p.R219W mutation on thermogenesis of female rodents. Given that quite a small number of p.R219W/Q carriers (*n* = 3) were found in male obese subjects, we next compared the clinical phenotypes only in female subjects (n = 9). We found that these female obese carriers showed a series of clinical features of obesity (age, 24.00 ± 2.76 years; BMI, 34.97 ± 0.94 kg m^−2^) and metabolic disorders compared to age‐ and sex‐matched healthy lean controls, but no significant changes in the metabolic parameters were found between obese carriers and non‐carriers (Table S5, Supporting Information).

At last, we isolated iWAT SVFs from *Rspo1* ^R219W^ and wild‐type mice, respectively, and induced them into mature beige adipocytes. We found that the mRNA expression of thermogenesis and mitochondrial respiration‐related genes was repressed in the beige adipocytes derived from the *Rspo1* ^R219W^ mice, which could be eliminated by the Wnt signaling blockade (Figure 6L). Based on these mouse and human data, we reveal for the first time at an organism level that the RSPO1 p.R219W mutation identified in young obese patients can suppress thermogenic capacity and lead to adiposity in obesogenic environments.

## 3 Discussion

Identifying functional BAT in adult humans has motivated obesity research in the clinical and molecular aspects of this long‐neglected tissue.^[^ ^1^, ^3^, ^32^ ^]^ Association studies in populations and biological experiments in animal models further revealed the therapeutic potential of increasing the thermogenic capacities of brown/beige adipocytes, which, however, are commonly repressed in obesity.^[^ ^3^ ^]^ According to a recent comprehensive review by Kajimura et al,^[^ ^6b^ ^]^ no clear evidence of human functional mutations that causally lead to severe adiposity through inhibiting thermogenesis has been reported in patients with obesity until now. We previously reported the genetic mutations and biological roles of Wnt/*β*‐catenin signaling molecules in the development of obesity.^[^ ^16^, ^28^, ^33^ ^]^ In this study, using systematic genetic screening of all known WNT genes as well as their paracrine activators and inhibitors, we reveal that a gain‐of‐function mutation in RSPO1 (p.R219W), which is enriched in young, severely obese subjects, inhibits the thermogenic capacities and mitochondrial respiration of brown/beige fat, reduces whole‐body energy expenditure, and results in adiposity in the homologous mutation knock‐in mouse model. Artificially overexpressing human RSPO1 in fat tissues also resulted in similar obesity phenotypes. As Rspo1 is preferentially expressed in the fibroblasts of eWAT‐derived SVFs, the mutation disrupts RSPO1's electrostatic binding to the ECM and its local concentration gradient, resulting in abnormal Wnt signaling activation in receptor‐enriched preadipocytes. These data suggest that RSPO1 functions as a novel endogenous suppressor of thermogenic adipocytes, and its genetic overactivation or overexpression contributes to the development of obesity likely in a fine‐tuned paracrine manner (Figure S10A,B, Supporting Information). Importantly, genetic ablation of *Rspo1* robustly increases the thermogenic capacity of visceral fat and resists HFD‐induced obesity in vivo, indicating the underlying therapeutic potential to treat obesity by blocking RSPO1 signaling in adipose tissues (Figure S10C, Supporting Information).

RSPO1–4 are paracrine‐secreted proteins and niche‐derived signals (morphogens) for stem cell maintenance and remodeling and tissue development, especially in the gastrointestinal system.^[^ ^16h^ ^]^ RSPO proteins contain an N‐terminal signal peptide (SP), two furin‐like domains (FRs), one thrombospondin protein 1 domain (TSP1) and a C‐terminal (CT) region enriched with positively charged amino acids that facilitate their electrostatic interactions with ECM components, such as HSPGs, and effectively preclude their long‐distance or random local diffusion.^[^ ^27a^ ^]^ However, RSPO1, RSPO2 and RSPO4 exhibit distinct and diverse roles in human diseases when homozygous loss‐of‐function mutations occur individually.^[^ ^22^, ^34^ ^]^ Homozygous loss‐of‐function RSPO1 mutations lead to a rare human syndrome that features 46, XX sex reversal, palmoplantar hyperkeratosis and a predisposition to squamous cell carcinoma of the skin,^[^ ^22^ ^]^ some of which were recapitulated in our *Rspo1* ^−/−^ mice, such as keratinized beads occurring in the skin of aged male *Rspo1* ^−/−^ mice and seminiferous tubules of the testis present in the ovary of female *Rspo1* ^−/−^ mice (data not shown). However, to the best of our knowledge, no gain‐of‐function mutations of these four genes have been reported in obese subjects; furthermore, few functional mutations in other paracrine proteins leading to diseases due to their disruption of physical binding to the ECM components have been disclosed.^[^ ^35^ ^]^ In this context, we propose a previously unknown “paracrine disorder model” in the development of obesity. A recent crystallographic study of RSPO3 revealed that substitution of the positively charged Arg residues (R216, R218, and R220; all neighboring the RSPO3 K219 residue, the homologous residue of RSPO1 R219) with negatively charged glutamic acid (Glu) residue in the CT region of RSPO3 damaged its heparin‐binding ability.^[^ ^27b^ ^]^ We identified p.R219W/Q mutations in RSPO1 that were enriched in the obese subjects (12 obese carriers, 0.62% of 1944 obese cases), did not occur in the functional domains (FR and TSP1) but presented in the CT domain. Interestingly, deletion of the CT domain was prevailingly considered to have no impact on the molecular function of the recombinant full‐length RSPO1 protein to activate the WNT pathway in vitro.^[^ ^36^ ^]^ But based on our findings, it is reasonable to assume that in vivo, the mutations lead to more release of RSPO1 protein into the intercellular space from ECM and overactivation of canonical Wnt signaling of target cells (like LGR4 positive preadipocytes), consequently preventing cell fate toward beige adipocytes. These results presumably indicate that the extracellular distribution or spatial patterns of RSPO1 are fine‐tuned to regulate Wnt activity in target cells, further supporting that local concentration gradients and the distance of paracrine Wnts are essential for body weight homeostasis.^[^ ^37^ ^]^ Cold exposure or *β*3‐adrenergic agonist stimulation decreases the fibrogenic profile of stromal vascular cells and promotes beige adipocyte function.^[^ ^12d^ ^]^ More recently, overexpression of PRDM16 in adipocytes reduces both HFD‐ and ageing‐induced fibrosis along with an enhanced browning program,^[^ ^12c,12d^ ^]^ implicating a negative regulation existed between fibroblast cells and beige adipocytes. Our evidence here indicates that RSPO1 in fibroblasts of WATs can respond to external cues (showing a robust decrease after coldness or *β*3‐adrenergic agonist stimulation) and act as a novel browning‐inhibitory factor that may mediate the inter‐cellular communications. As an understanding of how the ECM interacts with paracrine proteins is regarded as physiologically and pathologically important,^[^ ^14^, ^38^ ^]^ this RSPO1 mutation occurring in human obesity may provide us with a unique opportunity to re‐evaluate how morphogens move to organize the development of beige fat.^[^ ^14a^ ^]^

On the other hand, RSPO1–4 was recently identified as endogenous ligands of LGR4/5/6 receptors and ZNRF3/RNF43 E3 ligases,^[^ ^39^ ^]^ but they exhibit distinct capacities to potentiate canonical or noncanonical Wnt/*β*‐catenin signaling pathways.^[^ ^39a^ ^]^ In certain contexts, RSPO2/3 acts independently of LGR4/5/6.^[^ ^34c^ ^]^ The functional trajectories among RSPOs and their potential receptors in distinct biological processes and related diseases are still largely undetermined.^[^ ^40^ ^]^ LGR4/5/6 were initially classified as orphan G‐protein coupled receptors (GPCRs) for unknown ligands,^[^ ^41^ ^]^ and subsequent studies revealed that LGR4 but not LGR5/6 exerts various roles in infertility,^[^ ^42^ ^]^ osteoporosis,^[^ ^43^ ^]^ cancer,^[^ ^44^ ^]^ obesity^[^ ^33^, ^44^ ^]^ and cardiovascular disease.^[^ ^45^ ^]^ Common genetic variations in/around the genes encoding Wnt/*β*‐catenin signaling molecules, including TCF7L2, RSPO3, ZNRF3 and LGR4, are associated with obesity and cardiometabolic disorders,^[^ ^16^, ^46^ ^]^ but little pathogenic evidence of functional mutations, including gain‐of‐function or loss‐of‐function missense mutations, in these genes has been reported. Our previous studies revealed that a low‐frequency gain‐of‐function missense mutation in LGR4 (p.A750T) and rare gain‐of‐function missense mutations in CTNNB1/*β*‐catenin (p.T59A/p.R124H/p.R274H) are associated with a higher risk of obesity,^[^ ^16^, ^33^ ^]^ which warrants the potential involvement of genetic disorders of upstream signaling, including WNTs, WNT inhibitors and activators, in the development of obesity.^[^ ^16c^ ^]^ Together with this work, we anticipate that genetic and functional overactivation of the intact RSPO1–LGR4–CTNNB1/*β*‐catenin pathway regulates energy homeostasis and contributes to adiposity in humans.

In addition, visceral fat is inertly converted into beige fat compared to subcutaneous WAT, even in response to external strong cues such as long‐term coldness.^[^ ^2^ ^]^ Interestingly, we observed a fat depot‐dependent response to RSPO1 manipulation in vivo. *Rspo1* is much more abundantly expressed in eWAT than in iWAT and BAT. In this context, both overexpression of RSPO1 in adipose tissues (h*RSPO1* ^Tg^) and genetic over‐activation of p.R219W (*Rspo1* ^R219W^) profoundly attenuates the browning program of iWAT due to the low endogenous levels of *Rspo1* in iWAT. Conversely, the ablation of abundant endogenous *Rspo1* expression in eWAT robustly enhances the mitochondrial content and browning program of visceral fat. This phenomenon, to some extent, reveals the underlying molecular causes for the poorly browning capacity of visceral versus subcutaneous WAT.^[^ ^47^ ^]^ As a result, ablating *Rspo1* in eWAT significantly reduced the mass of eWAT and diet‐induced adiposity. Given the core roles of visceral fat in cardiometabolic disorders, attenuating RSPO1 might represent a potent modality to increase the thermogenic capacity of visceral WAT (vWAT), thereby combating central adiposity and its related disorders.^[^ ^2^ ^]^

In this study, we identified 12 obese cases carrying RSPO1 p.R219W/Q mutations. We also examined in these carriers the low‐frequency/rare variants in other reported genes that cause obesity in literature.^[^ ^48^ ^]^ We found that two cases additionally carried an SNP (rs1805094, p.K656N) in *LEPR* gene, and one case carried an SNP (rs2229616, p.V103I) in *MC4R* gene, while the others did not carry any combined mutations. Several studies have revealed that there is no significant association between the rs1805094 variant and adiposity,^[^ ^49^, ^50^ ^]^ while the rs2229616 variant protects from adiposity.^[^ ^51^ ^]^ Therefore, p.R219W/Q mutants found in these obese cases probably did not interact with other reported genetic variants of obesity. Of note, although these p.R219W/Q mutants showed a relatively high obesity risk (ORs, around 3.0 to 5.0), the penetrance of obesity in carriers with these mutations is incomplete, as some carriers from the control group or the probands’ pedigree retain the lean phenotype. Importantly, the humanized *Rspo1* ^R219W^ animal experiments and mechanistic studies provided evidence for the causal effect of this mutation on obesity. Future studies on more family studies are essential to reveal the clinical features and penetrance of these rare RSPO1 mutations. In addition, we observed more pronounced weight gain and thermogenesis suppression in female *Rspo1* ^R219W^ mice compared with wild‐type mice, but no significant changes were observed in male mice, indicating a possible sex‐by‐genotype interaction between RSPO1 p.R219W mutation and obesity. Similarly, the adipocyte‐specific genetic manipulation of *Tcf7l2*, a key component of Wnt signaling, also exhibits a sex‐specific difference in metabolic phenotypes.^[^ ^31^, ^52^ ^]^ A recent study showed that adipocyte‐specific overexpression of *Ndufv2*, a core subunit of Complex I, results in decreased adiposity and visceral fat content by increasing mitochondrial respiration, especially in female mice.^[^ ^53^ ^]^ Interestingly, we also observed a profound reduction of Complex I together with other components in female *Rspo1* ^R219W^ mice. However, we did not observe sex differences in the prevalence of p.R219W/Q mutation, and a larger population study will offer advantages to understanding whether a potential sex‐by‐genotype interaction exists in humans.

## 4 Conclusion

This study identifies a gain‐of‐function RSPO1 mutation (p.R219W) that acts as a genetic trigger to drive obesity in response to HFD feeding and reveals the possible etiology of human RSPO1 mutation in repressing thermogenic capacity by disrupting its electrostatic binding to ECM. Blocking RSPO1 signals promotes mitochondrial respiration and thermogenesis and reduces adiposity, highlighting its pivotal therapeutic implications for treating human obesity.

## 5 Experimental Section

### Human Participants

For genetic evaluation of rare and low‐frequency variants in the coding region of all WNTs, WNT activators, and WNT inhibitors, they analyzed their in‐house WES database consisting of 1,944 obese patients and 2,161 ethnically matched healthy lean control subjects was analyzed; cohort information and sequencing methods had been described in the authors’ previous studies.^[^ ^16^, ^18^ ^]^ In brief, obese patients were recruited from the Genetics of Obesity in Chinese Youngs (GOCY) study, which is registered at ClinicalTrials.gov (https://clinicaltrials.gov/ct2/show/NCT01084967). Patients with obesity were diagnosed at the specialized obesity outpatient clinic of Ruijin Hospital, Shanghai Jiao Tong University School of Medicine (SJTUSM). Secondary or syndromic obesity was clinically excluded. Anthropometric examinations, including measurements of weight, height, waist circumference (WC), hip circumference (HC), and blood pressure, were performed by experienced nurses according to a standard protocol. BMI was calculated as weight (kg)/[height (m)^2^].^[^ ^1b^ ^]^ The clinical procedures were performed according to previously described protocols.^[^ ^33^, ^54^ ^]^ Lean subjects were recruited as the control group from the authors’ previously established community population^[^ ^55^ ^]^ according to the following inclusion criteria: sex‐, nationality‐, and geography (Chinese ancestry from Southeast China)‐matched with the obese cases; 17.0 kg m^−2^ < BMI < 23.0 kg m^−2^; fasting blood glucose levels < 6.1 mmol L^−1^, 2‐hour postprandial blood glucose levels < 7.8 mmol L^−1^ with 75 g glucose‐oral glucose tolerance test, and HOMA‐IR < 2.5 in which the insulin resistance index (homeostasis model assessment of insulin resistance, HOMA‐IR) was defined as fasting insulin (IU/mL) × fasting glucose (mmol L^−1^)/22.5; blood pressure < 140/90 mmHg; 1.8 mmol L^−1^ < LDL‐cholesterol levels < 3.4 mmol L^−1^; ALT < 40 IU/L and AST < 40 IU/L; estimated glomerular filtration rate (eGFR) > 60 mL/min/1.73 m^2^; without clinically diagnosed metabolic diseases, and without use of anti‐diabetic, anti‐hypertensive and anti‐hyperlipidemic agents or weight‐loss drugs; without gastrointestinal diseases, cancer or cardiorespiratory dysfunction; without smoking history. WES was performed according to the authors’ previous studies.^[^ ^16^, ^18^ ^]^ By filtering for common variants with minor allele frequency (MAF) > 5% and synonymous variants, a list of rare and low‐frequency missense and indel variants was obtained that were significantly enriched in the case or control group for further analysis. Human adipose tissues were obtained during surgical procedures, for example, bariatric surgery and biliary tract surgery, followed by immediate storage in liquid nitrogen.^[^ ^33a^ ^]^ To evaluate the association between plasma RSPO1 levels and obesity, 452 participants, including 226 overweight/obese subjects (age, 34.79 ± 0.66 years, BMI, 32.47 ± 0.39 kg m^−2^) and 226 age‐ and sex‐matched lean subjects (age, 34.72 ± 0.66 years, BMI, 21.83 ± 0.13 kg m^−2^), were further recruited from a deep‐phenotype study of metabolic diseases at SAIC Volkswagen Automotive Company Limited, Shanghai (MedSV study).^[^ ^56^ ^]^ Plasma RSPO1 levels were determined using a commercial RSPO1 ELISA kit (R&D, DY4645‐05) according to the manufacturer's procedures. This study was approved by the Institutional Review Board of the Ruijin Hospital, Shanghai Jiao Tong University School of Medicine (SJTUSM) (2010‐6‐2) and was performed in accordance with the principle of the Helsinki Declaration II. Written informed consent was obtained from each participant.

### Mice

Adipose tissue‐specific h*RSPO1* transgenic (h*RSPO1* ^Tg^) mice, global *Rspo1* knockout (*Rspo1* ^−/−^) mice, and humanized Rspo1 p.R219W (*Rspo1* ^R219W^) mice were first generated by a commercial provider (Cyagen). In brief, for the h*RSPO1* ^Tg^ mouse model, human *RSPO1* cDNA (ORF022949) was cloned downstream of the *aP2* promoter (aP2>hRSPO1). Gel‐purified linear fragments were microinjected into fertilized C57BL/6 mouse oocytes and transferred to pseudopregnant females. The transgenic mice were screened by PCR using primers that specifically detected h*RSPO1*: forward primer, GAGGTGGATTCAAGCAGGACAG; reverse primer, GGGTCAGTGGCAGGAGAGC. *Rspo1* ^−/−^ mice were produced on a C57BL/6 mouse background by microinjection of TALENs in fertilized eggs. Exon 3 in the *Rspo1* gene was selected as the TALEN target site with the following sequences: TALEN‐L, TGAGCTCTGTTCAGAAGT; TALEN‐R, TGAAGAGCTTGGGCGAGC; Spacer, CAACGGTTGCCTCAAGT. TALENs were constructed using the Golden Gate Assembly method and confirmed by sequencing. TALEN mRNA, generated by in vitro transcription, was injected into fertilized eggs for the knockout mouse production. The pups were genotyped by PCR, followed by sequencing analysis with specific primers: CTCAAGTGCTCGCCCAAGCTCTT and ATTTGTTCATGTCGGGGTTGCGG. 10‐bp nucleotides were confirmed to be deleted by Sanger sequencing, which led to a frameshift mutation and disrupted Rspo1 protein function. Wild‐type littermates were used as controls for h*RSPO1* ^Tg^ and *Rspo1* ^−/−^ mice, respectively. *Lgr4* ^m/m^ and*ob*/*ob* mice were bred as described in the authors’ previous study.^[^ ^33a^ ^]^ *Rspo1* ^R219W^ knock‐in mice were created using CRISPR/Cas9‐mediated genome engineering. Exon 6 of the *Rspo1* gene was selected as the target site. A gRNA targeting vector (matching reverse strand of gene: GCCTGTTGGCATTCTCCCTCCGG) and donor oligo (targeting sequence: TTGATTTTCCTCCACTATCCAACTGTTGCAGGGCAGAAGAGGAGGAAGGGGGGCCAGGGCTGGAGGGAGAATGCCAACAGGCATCCGGCCAGGAAGAACAGCAAGGAGCCGGGCTCCAACTCT, flanked by 120‐bp homologous sequences combined on both sides) were designed. The p.R219W (CGG to TGG) in the donor oligo was introduced into exon 6 by homology‐directed repair. Cas9, gRNA, and the donor oligo were coinjected into fertilized eggs for the knock‐in mouse production. The pups were genotyped by PCR followed by sequencing analysis: forward primer, AGGAAATGCTCTAGGGTCTTTAG; reverse primer, GAATAGAAGTGTCTTCAGTGACC. The p.R219W mutation created in the mouse *Rspo1* gene was homologous to the p.R219W mutation in the human *RSPO1* gene. Mice were maintained on a 12‐hour light‐dark cycle and fed ad libitum.

A whole‐body composition analyzer (EchoMRI) was used to measure body composition in awake animals. For energy expenditure measurement, mice were placed in metabolic cages (Columbus Instruments generally for room temperature tests or Sable Systems International generally for cold stimulation tests) to assess their heat production, O_2_ consumption, CO_2_ production and physical activity. Rectal temperature was measured with a model BAT‐12 thermometer (Physitemp Instruments). To determine energy expenditure in response to the cold challenge, the homozygous *Rspo1* ^R219W^ knock‐in mice and their wild‐type littermates were placed into cages individually. After two days of adaptation to room temperature (22 °C), basal energy expenditure data were collected, and then the cage temperature was cooled to 4 °C for data collection in the next 24 h. In HFD experiments, mice were fed a 60 kcal% HFD (Research Diet, 12492i), usually starting at 8 weeks. In some experiments, mice were injected with the *β*3‐adrenergic receptor agonist CL316,243 (Sigma) at 1 mg kg^−1^ for three or seven days, or mice were subjected to 4 °C cold room stress for ten days before samples and data were collected.^[^ ^54^ ^]^ Nutrient absorption was quantified as described previously. Fecal pellets were collected daily from the cage floor for 3 consecutive days, during which time mice were single‐caged. Fecal pellets were dehydrated and then subjected to bomb calorimetry using a Parr 6725 Semimicro Calorimeter. All procedures were approved by the Animal Care Committee of SJUSM.

### Cell Culture and Transfection

HEK293T cells were obtained from American Type Culture Collection (ATCC) and maintained in Dulbecco's modified Eagle's medium (DMEM) supplemented with 10% (v/v) fetal bovine serum (FBS) (Gibco) at 37 °C in a 5% CO_2_ humidified atmosphere. Cell transfection was performed using Lipofectamine 2000 (Invitrogen) according to the manufacturer's instructions.

### Plasmids and Luciferase Reporter Assay

The coding region of RSPO1 was amplified from a human cDNA clone (NM_001038633) and was subcloned into the pcDNA3.1(+)‐IRES‐GFP vector (N‐terminal flag‐tag) between the Nhel and BamHI restriction sites. All 18 RSPO1 mutations (Table 1) were generated using the QuickChange Site‐Directed Mutagenesis Kit (Stratagene) according to the manufacturer's instructions. Full‐length coding sequences for all plasmids were verified by DNA sequencing. Plasmids encoding the *β*‐catenin and TOP‐Flash had been previously described.^[^ ^16f^ ^]^ For the luciferase reporter assay, HEK293T cells were transfected with TOP‐Flash plasmid, SV40 (expressing Renilla luciferase for normalization), and human wild‐type or mutant RSPO1 plasmids with indicated dosages in 24‐well or 96‐well plates for 24 h. Cells were harvested after an additional 12‐hour culture with PBS or 100 ng mL^−1^ Wnt3a (R&D, 5036‐WN‐010), followed by luciferase activity measurement using a dual‐luciferase reporter assay system (Promega, E1980) or the nuclear translocation assessment of *β*‐catenin protein using Western blotting. For neutralization experiments, cultured media harvested from HEK293T cells transfected with wild‐type or RSPO1 mutant plasmids were added to the TOP‐Flash reporter system in the presence or absence of 2 µg mL^−1^ RSPO1 neutralizing antibody (R&D, MAB3474).

### Protein Preparation and Western Blotting

Proteins from adipose tissues, and primary or culture cells were generally isolated with cold radioimmunoprecipitation assay (RIPA) buffer, followed by concentration quantification with the BCA Protein Assay kit (Pierce Biotechnology, 23227). SVF cells from wild‐type and *Lgr4* ^m/m^ mice were treated with 100 ng mL^−1^ recombinant human RSPO1 protein (hRSPO1) (R&D, 4645‐RS), 100 ng mL^−1^ Wnt3a or their combination. After 12‐hour treatment, the nuclear and cytoplasmic protein extracts were isolated using a nuclear and cytoplasmic extraction kit according to the manufacturer's protocol (CWBIO, cw0199). For the detection of thermogenic proteins in differentiated mature beige adipocytes, SVF cells from wild‐type and *Lgr4* ^m/m^ mice were induced with or without 100 ng mL^−1^ hRSPO1 treatment, or SVF cells from wild‐type mice were induced with or without the treatment of 100 ng mL^−1^ hRSPO1 and/or 5 µm IWR‐endo1 (Selleck). For the detection of RSPO1 protein levels in different components of cultured cells, HEK293T cells were transfected with human wild‐type or mutant RSPO1 (p.R219W/Q) plasmids. 24 h after transfection, the culture medium was replaced with fresh serum‐free medium and then supplemented with PBS or 50 µg mL^−1^ heparin (Qilu). Conditioned media and cell pellets were harvested after an additional 12‐hour culture. The separation of three components, cell lysates, ECM, and conditioned medium (CM) fractions, was performed as previously described.^[^ ^57^ ^]^ For the assessment of RSPO1 protein binding to HS molecule, cell lysates from HEK293T cells transfected with flagged wild‐type RSPO1 were collected to incubate with HS beads, which were then eluted by high‐concentration salt solution (NaCl, 1.2 m). Anti‐flag antibody was applied to detect the flagged RSPO1 levels in cell lysates, HS beads before and after elution, and the eluted solution, respectively. In further, after CM were collected from HEK293T cells transfected with plasmids of flagged wild‐type or RSPO1 p.R219Q/W mutants, a proportion of CM (5 µL) was reserved as input (CM Input) to detect total RSPO1 levels, and the other part was incubated with HS‐beads to collect pull‐down materials (HS Pull‐down). Proteins were denatured in boiling water, separated by 10% or 12.5% SDS–PAGE, and transferred onto nitrocellulose membranes (Amersham Biosciences). After blocking in milk for 1 h, the membranes were incubated at 4 °C overnight with the primary antibodies. Detailed information on the antibodies used in this study is provided in Table S6, Supporting Information. The resulting bands were visualized using a luminescent image analyzer (GE, ImageQuant LAS400).

### Morphological Analysis

Tissues were sampled and fixed in 4% neutral buffered formalin and then embedded in paraffin. Sections of 5 µm thickness were stained with hematoxylin and eosin (H&E) according to standard protocols. Immunohistochemical staining of UCP1 was performed using an anti‐UCP1 antibody (1:500, Abcam, ab10983). Immunofluorescence staining was performed with an anti‐UCP1 antibody and an anti‐Perilipin antibody (1:500, Cell Signaling Technology, CS9349s), respectively, and the slides were mounted with DAPI (4′,6‐diamidino‐2‐phenylindole) Fluoromount‐G mounting media (Southern Biotech). Images were acquired by microscopy (TissueFAXS PLUS). All representative images were repeated in at least three mice. Electron microscopy of WAT was performed in accordance with the authors’ previous study.^[^ ^33a^ ^]^

### SVF Isolation, Mature Adipocyte Isolation, Beige Adipocyte Differentiation, and Oil Red O Staining

Mouse SVFs were isolated as described in the authors’ previous study.^[^ ^33a^ ^]^ In brief, adipose tissues were minced and digested in 2 mg mL^−1^ collagenase type II (Sigma) in HEPES (Invitrogen) supplemented with 1% bovine serum albumin for 30 min at 37 °C, followed by quenching with complete medium. Cell suspensions were centrifuged, washed, and filtered through a 40‐µm strainer (BD Biosciences) and then either plated on 6‐cm dishes for induction. In some experiments, mature adipocytes (white ring) floating in the top part of the resuspension were carefully aspirated and transferred into a 50 mL tube, washed with 5 mL DMEM/F12 medium, and centrifuged at 1000 rpm for 5 min twice. Cells were cultured in DMEM supplemented with 10% fetal bovine serum (FBS) (Gibco), 1% penicillin/streptomycin (Invitrogen), and 10 ng mL^−1^ murine basic FGF (R&D). SVF cells were then plated onto 48‐well plates and allowed to reach confluence. Beige adipocyte differentiation was induced using a cocktail medium containing 5 µg mL^−1^ insulin (Eli Lilly), 0.5 mm isobutylmethylxanthine (Sigma), 1 µm dexamethasone (Sigma), 1 nM T3 (Sigma), and 1 µm troglitazone (Sigma) for 48 h followed by the addition of growth medium supplemented with insulin, T3 and rosiglitazone for six days. A specific Rspo1‐shRNA lentivirus was constructed and was applied to knock down endogenous Rspo1 expression in the SVF cells before induction. The sequence of mouse Rspo1 lentiviral shRNA is provided in Table S7. Supporting Information. For Oil Red O staining, fully differentiated beige adipocytes were fixed in 4% paraformaldehyde for 20 min, followed by Oil Red O (Sigma) incubation for 30 min. For Wnt pathway inhibitor treatment in beige adipocytes derived from *Rspo1* ^R219W^ mice, 5 µm IWR‐endo1 was added to the induction medium.

### OCR Measurements

SVFs were plated in an XF24 microplate (Seahorse Bioscience) coated with poly‐L‐lysine. With the exogenous hRSPO1 treatment or endogenous Rspo1 knockdown, the cells were induced into beige adipocytes for eight days, followed by OCR measurement at 37 °C using an XF24 analyzer (Seahorse Bioscience) according to the manufacturer's instructions. One micromolar oligomycin, 1.5 µm FCCP and 1.25 µm rotenone/antimycin were added to detect uncoupled, maximal, and non‐mitochondrial respiration, respectively.

### RNA Isolation and Real‐Time PCR

Total RNA was extracted from cells or adipose tissues using TRIzol reagent (Invitrogen) according to the manufacturer's protocols. One microgram of RNA was transcribed into cDNA using the Reverse Transcription System (Promega). Real‐time PCR was performed on an ABI system (Life Technology) using SYBR Green Supermix (Takara). The primers used in this study are provided in Table S7, Supporting Information. Data were normalized to 36B4 and analyzed using the ΔΔCT method.

### Single‐Cell Sequencing and Analysis

Raw sequencing data of mouse adipose tissues were obtained from the Sequence Read Archive (SRP145475)^[^ ^24^ ^]^ and preprocessed using Cell Ranger Single Cell Software to generate gene expression matrices. Two libraries, eWAT‐Sham‐Lin^−^ (SRR7154853) and iWAT‐Sham‐Lin^−^ (SRR7154857), were combined to identify different gene distributions between eWAT and iWAT. The combined matrix was then input into the open‐source R package Seurat^[^ ^58^ ^]^ to generate R objects under the setting of minimal cells equal to 3 for each feature and minimal features equal to 200 for each cell. K‐means clustering analysis was performed on the tSNE‐reduced data under the default setting of Seurat. The cluster identities were determined by matching the enriched genes of each cluster to a previous study.^[^ ^24^ ^]^ Violin plots and gene distribution plots were generated using the VlnPlot and FeaturePlot commands, respectively, within the Seurat package. The human single‐cell matrix was obtained from the Gene Expression Omnibus (GEO) with accession number GSE136230^[^ ^25^ ^]^ and used to generate Seurat objects to perform tSNE reduction with the same setting as for the mouse data. RSPO1‐positive cells were presented by highlighting the cells in which RSPO1 expression was larger than zero.

### Bulk‐RNA Sequencing and Analysis

Total RNA was extracted using a TRIzol reagent kit (Invitrogen). RNA quality was assessed on an Agilent 2100 Bioanalyzer (Agilent Technologies) and RNase‐free agarose gel electrophoresis. Eukaryotic mRNA was enriched by Oligo(dT) beads, fragmented into short fragments, and reversely transcribed into cDNA using NEBNext Ultra RNA Library Prep Kit for Illumina (New England Biolabs, NEB #7530). The purified double‐stranded cDNA fragments were end‐repaired, a base added and ligated to Illumina sequencing adapters, which were then subjected to size selection. The resulting cDNA library was sequenced using Illumina Novaseq6000. The procedure of data processing and analysis followed the methods described previously.^[^ ^59^ ^]^ In brief, faspt (version 0.18.0) was used to remove low‐quality reads and short reads and applied the alignment tool Bowtie2 (version 2.2.8) to map reads to the ribosome RNA (rRNA) database. The remaining clean reads were further mapped to the reference genome using HISAT2. 2.4 and other parameters were set as a default. Differential expression analysis between groups was performed by DESeq2 software.^[^ ^60^ ^]^ The transcripts with the parameter of false discovery rate (FDR) below 0.05 were considered as differentially expressed transcripts. Heat maps were generated using ComplexHeatmap package^[^ ^61^ ^]^ in R based on DESeq‐normalized reads counts of either differentially expressed genes between groups or genes listed in corresponding Reactome gene sets. All multiple comparisons were corrected by using the Benjamini–Hochberg method. GSEA was performed based on the GO:BP database.^[^ ^62^ ^]^ Ranked‐log_2_ folder change of all genes between RSPO1‐manipulated mice versus their wild‐type littermates were used to calculate the normalized enrichment score (NES) and FDR of each geneset in GO:BP, FDR below 0.1 was considered as statistical significance for GSEA results.

### Statistical Analysis

Summary data were all presented as the mean ± sem. For unrepeated continuous variables, unpaired Student's *t*‐test was used to identify the differences between groups. For genetic data, Chi‐square test was used to estimate the crude OR and 95% CI of each RSPO1 variant's frequency. And a logistic regression model was applied to assess the sex‐adjusted OR and genotype‐by‐sex interaction. For the repeated measurements of indirect calorimetry, hourly data were analyzed using a two‐way ANOVA model with the interaction between genotype and time followed by a pairwised *t*‐test with Benjamini–Hochberg correction as post‐hoc test. And the 24‐hour total indirect calorimetric measurements were assessed with ANCOVA model with genotype as the independent variable and body weight as the covariate if the body weight between genotypes had diverged before the examination.^[^ ^63^ ^]^ Microarray data that identified the expression distributions of Rspo1–4 were normalized across all Rspo family members and tissues before performing heat map analysis. All statistical analyses were performed using the open‐source software R. For each figure, *n* equals the number of independent biological replicates. Significant differences emerging from the above tests are indicated in the figures by *p < 0.05, **p < 0.01, ***p < 0.001; ns, not significant (*p* > 0.05).

## Conflict of Interest

The authors declare no conflict of interest.

## Author Contributions

J.W. conceptualized and designed the study. J.W., R.L., and W.W. supervised the study. Y.S., J.Z., J.H., Z.Z., and P.L. contributed equally to this work. The authors thank all the participants for their involvement in this study. Y.S., J.Z., P.L., and Z.Z. carried out animal and molecular experiments. J.W., J.S., H.Y., and Y.S. analyzed the genetic data. J.W., R.L., J.H., and Y.S. analyzed the clinical and experimental data. J.Z., Z.Z., A.G., M.N., Z.Z., J.L., W.X., Q.L., J.H., W.G., Y.B., and W.W. provided the cohort resources. J.W., R.L., Y.S., and J.Z. wrote the manuscript. W.W., G.N., and Y.Z. contributed to text revision and discussion.

## Supporting information

Supporting Information

## Data Availability

The data that support the findings of this study are available from the corresponding author upon reasonable request.
