# Supplementary material for: Human RSPO1 Mutation Represses Beige Adipocyte Thermogenesis and Contributes to Diet‐Induced Adiposity
Source: Adv Sci (Weinh). 2023 Feb 8;10(12):2207152. doi: 10.1002/advs.202207152 (PMC10131814; doi:10.1002/advs.202207152)
Supplement: Supplementary file 1 — Supporting Information [file ADVS-10-2207152-s001.pdf]

Supporting Information

Human RSPO1 Mutation Represses Beige Adipocyte Thermogenesis and Contributes to Diet-induced Adiposity

Yingkai Sun, Juan Zhang, Jie Hong, Zhongyun Zhang, Peng Lu, Aibo Gao, Mengshan Ni, Zhiyin Zhang, Huanjie Yang, Juan Shen, Jieli Lu, Wenzhi Xue, Qianqian Lv, Yufang Bi, Yi Arial Zeng, Weiqiong Gu, Guang Ning, Weiqing Wang, Ruixin Liu*, Jiqiu Wang**

Table S1. The 39 WNT-related secreted genes are genetically analyzed with whole-exome sequencing in GOCY study.

	Official names	Functional classification		Official names	Functional classification		Official names	Functional classification
**1**	WNT1	**WNTs**	**1**	DKK1	**WNT inhibitors**	**1**	NDP	**WNT activators**
**2**	WNT2		**2**	DKK2		**2**	RSPO1	
**3**	WNT2B		**3**	DKK3		**3**	RSPO2	
**4**	WNT3		**4**	DKK4		**4**	RSPO3	
**5**	WNT3A		**5**	SFRP1		**5**	RSPO4	
**6**	WNT4		**6**	SFRP2		**6**	SOSTDC1	
**7**	WNT5A		**7**	SFRP3				
**8**	WNT5B		**8**	SFRP4				
**9**	WNT6		**9**	SFRP5				
**10**	WNT7A		**10**	WIF1				
**11**	WNT7B		**11**	SOST				
**12**	WNT8A		**12**	CER1				
**13**	WNT8B		**13**	IGFBP4				
**14**	WNT9A		**14**	NOTUM				
**15**	WNT9B							
**16**	WNT10A							
**17**	WNT10B							
**18**	WNT11							
**19**	WNT16							

GOCY study, the **G**enetics of **O**besity in **C**hinese **Y**oungs (GOCY) study registered at ClinicalTrials.gov (NCT01084967).

Table S2. Four nonsynonymous variants among 39 genes are significantly associated with adiposity or lean phenotype.

Functional Classification	Gene	Position	Nucleotide change	Amino acid change	Case genotype (N=1944)	Control genotype (N=2161)	OR (95% CI)	P value	Adjusted-OR (95% CI)	P.adj
WNT activators	RSPO1	1:38078564	G>A	p.R219W	0/11/1933	0/3/2158	4.09 (1.08, 22.88)	0.0286	2.89 (1.16, 7.18)	0.0224
WNT inhibitors	SFRP5	10:99527530	G>T	p.P232Q	0/9/1935	0/25/2136	0.4 (0.16, 0.88)	0.0154	0.56 (0.32, 0.96)	0.0364
WNTs	WNT10A	2:219754840	C>T	p.R171C	1/61/1882	0/43/2118	1.62 (1.08, 2.47)	0.0172	1.48 (1.12, 1.97)	0.0061
WNT activators	RSPO4	20:948700	A>T	p.L54H	0/0/1944	0/9/2152	0 (0, 0.56)	0.00427	0 (0, Inf)	0.9383

* NCBI Build 37. †Variations are based on RefSeq records NM_001029871 and NP_001025042 for RSPO4, NM_003015 and NP_003006 for SFRP5, NM_025216 and NP_079492 for WNT10A, and NM_001038633 and NP_001033722 for RSPO1.

Table S3. Rare and low-frequency RSPO1 nonsynonymous variants identified in young Chinese with obesity and East Asian controls.

Position*	rsID	Nucleotide change†	AA change†	Cases	East Asian	All gnomAD	Case _freq	East Asian _freq	All gnomAD _freq	*P* value$	OR (95% CI)$	SIFT	Polyphen2 _HDIV
1:38095329	rs377442675	C>T	R2Q	2/1942	0/8985	2/124419	0.00051	0	0.00001	0.03	Inf. (0.87, Inf.)	T	B
1:38095278	-	A>T	I19N	0/1944	0/NA	0/NA	0	NA	NA	-	-	T	B
1:38095269	rs776558926	C>T	R22Q	0/1944	0/8989	14/124674	0	0	0.00006	1.00	0.00 (0.00, Inf.)	T	D
1:38095243	rs772445254	G>A	R31W	0/1944	6/8989	16/124552	0	0.00033	0.00006	0.60	0.00 (0.00, 3.93)	D	D
1:38082222	rs200218812	C>T	V74I	1/1943	0/8983	6/124536	0.00026	0	0.00002	0.18	Inf. (0.12, Inf.)	T	B
1:38082212	rs367943248	G>A	P77L	1/1943	7/9766	14/140183	0.00026	0.00036	0.00005	1.00	0.72 (0.02, 5.59)	T	D
1:38082174	rs996524953	C>T	D90N	1/1943	0/9764	4/139671	0.00026	0	0.00001	0.17	Inf. (0.13, Inf.)	D	D
1:38082163	rs1025272837	C>A	K93N	1/1943	0/NA	0/NA	0.00026	NA	NA	-	-	D	D
1:38079564	rs761409987	G>A	A146V	0/1944	0/9765	3/140280	0	0	0.00001	1.00	0.00 (0.00, Inf.)	T	B
1:38079517	rs36043533	T>G	K162Q	115/ 1829	544/ 9762	14512/ 140352	0.02984	0.02786	0.04982	0.23	1.14 (0.92, 1.40)	D	P
1:38079496	rs374906144	G>A	R169W	0/1944	1/9762	6/140317	0	0.00005	0.00002	1.00	0.00 (0.00, 195.25)	D	D
1:38078593	rs534011478	C>T	G209E	2/1942	11/9768	14/140285	0.00051	0.00056	0.00005	1.00	0.91 (0.10, 4.19)	T	D
1:38078590	-	T>G	Q210P	1/1943	0/NA	0/NA	0.00026	NA	NA	-	-	T	B
1:38078582	-	T>A	R213W	0/1944	0/NA	0/NA	0	NA	NA	-	-	D	D
**1:38078564**	**rs749116749**	**G>A**	**R219W**	**11/1933**	**3/9768**	**8/140412**	**0.00283**	**0.00015**	**0.00003**	**5.45×10^{-7}**	**18.6 (4.91, 104.18)**	**D**	**D**
**1:38078563**	**rs372057847**	**C>T**	**R219Q**	**1/1943**	**0/9769**	**20/140415**	**0.00026**	**0**	**0.00007**	**0.17**	**Inf. (0.13, Inf.)**	**D**	**P**
1:38078510	rs760850808	C>T	A237T	0/1944	1/8988	1/124650	0	0.00006	0	1.00	0.00 (0.00, 179.81)	T	B
1:38078500	rs61748625	C>T	R240Q	1/1943	0/9768	495/140305	0.00026	0	0.00176	0.17	Inf. (0.13, Inf.)	T	B

[*] NCBI Build 37. [†] Variations are based on RefSeq records NM_001038633 and NP_001033722, respectively. Freq, allele frequency. [$] cases versus East Asian controls. The data of East Asian and all gnomAD samples are from gnomAD v2.1.1 (https://gnomad.broadinstitute.org/gene/). Inf., infinity. NA, not available. Functional prediction is conducted by SIFT (https://sift.bii.a-star.edu.sg/) (T, Tolerated; D, Damaging), and PolyPhen2 (http://genetics.bwh.harvard.edu/pph2/) (B, Benign; D, Probably damaging; P, Possibly damaging).

Table S4. The plasma RSPO1 levels in normal weight controls and age- and sex-matched overweight/obesity cases.

	Normal weight	Overweight/Obesity	*P* value
N	226	226	
Age (year)	34.72 ± 0.66	34.79 ± 0.66	ns
Female:Male	80:146	80:146	ns
BMI (kg/m2)	21.83 ± 0.13	32.47 ± 0.39	***
WC (cm)	79.51 ± 0.51	105.52 ± 0.94	***
WHR	0.86 ± 0.01	0.94 ± 0.00	***
Fat (%)	23.70 ± 0.42	34.78 ± 0.54	***
SAT Volume (ml)	3847.79 ± 92.89	8440.94 ± 224.45	***
VAT Volume (ml)	2050.08 ± 73.28	4936.22 ± 105.14	***
HBA1C (%)	5.43 ± 0.06	5.72 ± 0.06	***
FPG (mmol/l)	5.39 ± 0.09	5.74 ± 0.07	**
2hPG (mmol/l)	6.31 ± 0.23	7.64 ± 0.21	***
FPI (μIU/ml)	8.69 ± 0.42	19.46 ± 1.43	***
2hPI (μIU/ml)	42.21 ± 1.92	102.88 ± 6.06	***
TG (mmol/L)	1.30 ± 0.07	2.08 ± 0.12	***
TC (mmol/L)	4.94 ± 0.07	5.08 ± 0.08	ns
HDL-c (mmol/L)	2.94 ± 0.06	3.20 ± 0.07	***
LDL-c (mmol/L)	1.49 ± 0.02	1.16 ± 0.02	**
RSPO1 (pg/ml)	57.33 ± 3.87	51.66 ± 3.88	ns

Data shown as mean ± SEM. ns, not significant; **, $P < 0.01$; ***, $P < 0.001$, between normal weight controls versus overweight/obese cases. BMI, body mass index. WC, waist circumference. WHR, waist-to-hip ratio. FBG and 2hPGB, blood glucose levels at 0 and 120 minutes of OGTT. FPI and 2hPI, plasma insulin levels at 0 and 120 minutes of OGTT. TG, triglyceride. TC, total cholesterol. HDL-c, HDL-cholesterol. LDL-c, LDL-cholesterol.

Table S5. The clinical parameters related to obesity in RSPO1 p.R219W/Q variant obese carriers and non-carriers, and lean healthy controls.

Clinical parameters	Normal controls		R219W/Q variant obese carriers		non-R219W/Q variant-carried obese subjects	
	N	Mean ± SEM	N	Mean ± SEM	N	Mean ± SEM
Age (year)	239	23.11 ± 0.23	9	24.00 ± 2.76	1034	24.02 ± 0.21[&]
Height (m)	224	1.62 ± 0.00	9	1.64 ± 0.01	1024	1.64 ± 0.00[&]
Weight (kg)	221	53.15 ± 0.31	9	93.52 ± 1.94[*]	1022	94.70 ± 0.48[&]
BMI (kg/m^2)	233	20.31 ± 0.08	9	34.97 ± 0.94[*]	1017	35.25 ± 0.16[&]
WC (cm)	228	70.44 ± 0.34	9	106.17 ± 2.6[*]	973	106.58 ± 0.38[&]
HC (cm)	222	90.67 ± 0.29	9	116.71 ± 3.00[*]	973	115.17 ± 0.31[&]
WHR	221	0.78 ± 0.00	9	0.91 ± 0.02[*]	972	0.93 ± 0.00[&]
HBA1C (%)	134	5.14 ± 0.03	5	5.68 ± 0.04[*]	711	5.97 ± 0.12[&]
FPG (mmol/l)	239	4.68 ± 0.02	9	4.88 ± 0.09	991	5.48 ± 0.05[&]
2hPG (mmol/l)	233	5.41 ± 0.07	9	7.88 ± 0.45[*]	989	8.26 ± 0.11[&]
FPI (μIU/ml)	239	7.10 ± 0.23	9	20.02 ± 2.26[*]	981	25.34 ± 0.78[&]
2hPI (μIU/ml)	193	41.6 ± 1.8	9	194.61 ± 33.99[*]	978	162.16 ± 4.21[&]
TG (mmol/l)	227	0.78 ± 0.02	7	1.32 ± 0.16[*]	898	1.62 ± 0.04[&]
TC (mmol/l)	228	4.41 ± 0.2	7	4.74 ± 0.39	896	4.60 ± 0.03
HDL-c (mmol/l)	227	1.57 ± 0.02	7	1.09 ± 0.08[*]	898	1.16 ± 0.02[&]
LDL-c (mmol/l)	228	2.27 ± 0.04	7	2.98 ± 0.32[*]	896	2.86 ± 0.02[&]
ALT (IU/L)	220	15.12 ± 0.46	8	50.88 ± 14.88[*]	936	45.5 ± 1.34[&]
AST (IU/L)	220	18.99 ± 0.29	8	32.00 ± 7.44	937	30.93 ± 0.75[&]
GGT (IU/L)	219	11.54 ± 0.24	7	31.29 ± 9.09	932	30.52 ± 0.95[&]

All subjects involved in this analysis are female participants. [*], $P < 0.05$ between normal controls versus R219W/Q variant obese carriers; [&], $P < 0.05$ between normal controls versus non-R219W/Q variant-carried obese subjects. No significance was found between carriers and non-carriers of subjects with obesity. BMI, body mass index. WC, waist circumference. HC, hip circumference. WHR, waist-to-hip ratio. FBG and 2hPGB, blood glucose levels at 0 and 120 minutes of OGTT. FPI and 2hPI, plasma insulin levels at 0 and 120 minutes of OGTT. TG, triglyceride. TC, total cholesterol. HDL-c, HDL-cholesterol. LDL-c, LDL-cholesterol. ALT, alanine aminotransferase. AST, aspartate aminotransferase. GGT, gamma glutamyl transpeptidase.

Table S6. The information of Western blotting antibodies used in this study.

Antibodies	Sources	Dilution for WB	Catalogy No.
Anti-Flag	Beyotime Biotechnology	1:1000	AF519
Anti-HSP90	Cell Signaling Technology	1:1000	4877s
Anti-UCP1	Alpha Diagnostic	1:1000	UCP11-A
Anti-OXPHOS	Abcam	1:1000	ab110413
Anti-UCP1	Abcam	1:1000	ab10983
Anti-PGC-1α	Millipore	1:1000	ab3242
Anti-β-catenin	Cell Signaling Technology	1:1000	9562
Anti-α-Tubulin	Cell Signaling Technology	1:1000	2144S
Anti-Collagen V	Abcam	1:1000	ab7046
Anti-GAPDH	Cell Signaling Technology	1:1000	5174
Anti-HistonH3	Cell Signaling Technology	1:2000	4499
Anti-rabbit IgG	Cell Signaling Technology	1:1000	7074s
Anti-goat IgG	Santa Cruz Biotechnology	1:1000	SC2033
Anti-mouse IgG	Cell Signaling Technology	1:1000	7076s
Anti-LGR4	Sigma	1:1000	HPA030267
Anti-LGR5	Abcam	1:500	75850
Anti-LGR6	Santa Cruz Biotechnology	1:500	393010
Anti-ZNRF3	Biomol	1:1000	CAB16026.100
Anti-RNF43	Abcam	1:500	84125
Anti-FABP4	Santa Cruz Biotechnology	1:500	SC271529
Anti-PPARγ	Santa Cruz Biotechnology	1:1000	SC7273

Table S7. Sequence information of real-time PCR primers and Rspo1-shRNA applied in the study.

Genes	Forward primer sequence	Reverse primer sequence
mRspo1	GGGATCAAGGGCAAGAGACAG	CTGGCGGATGTCGTTCCTC
mUcp1	AGGCTTCCAGTACCATTAGGT	CTGAGTGAGGCAAAGCTGATTT
mPgc-1α	TATGGAGTGACATAGAGTGTGCT	CCACTTCAATCCACCCAGAAAG
mCidea	TGACATTCATGGGATTGCAGAC	GGCCAGTTGTGATGACTAAGAC
mDio2	CAGTGTGGTGCACGTCTCCAATC	TGAACCAAAGTTGACCACCAG
mCox7a1	GCTCTGGTCCGGTCTTTTAGC	GTACTGGGAGGTCATTGTCGG
mCox8b	TGTGGGGATCTCAGCCATAGT	AGTGGGCTAAGACCCATCCTG
mNdufa8	AAGTGGAGGAGGTGAAAGTCAG	GTTTTATCGCACTGAGCCCC
mUqcrc2	AAAGTTGCCCCGAAGGTTAAA	GAGCATAGTTTTCCAGAGAAGCA
mRspo2	CCAAGGCAACCGATGGAGAC	TCGGCTGCAACCATTGTCC
mRspo3	ATGCACTTGCGACTGATTTCT	GCAGCCTTGACTGACATTAGGAT
mRspo4	CTCGCCCTGTACCGAAGGA	CACTTGCCGTACTGACGGATG
mPrdm16	CCAAGGCAAGGGCGAAGAA	AGTCTGGTGGGATTGGAATGT
mPparγ	TCGCTGATGCACTGCCTATG	GAGAGGTCCACAGAGCTGATT
m36b4	AGATTCGGGATATGCTGTTGGC	TCGGGTCCTAGACCAGTGTTC
m-β-actin	ACTCCAAGGCCACTTATCACC	ATTGTTACCAACTGGGACGACA
mβ-catenin	ATGGAGCCGGACAGAAAAGC	TGGGAGGTGTCAACATCTTCTT
mLgr4	TTGTGGGTAACTTCCAGCTGA	GTGGCATTTGCTGCATCTGTA
mLgr5	CCTACTCGAAGACTTACCCAGT	GCATTGGGGTGAATGATAGCA
mLgr6	GAGGACGGCATCATGCTGTC	GCTCCGTGAGGTTGTTCATACT
mZnrf3	CACTGTCGGCACAACATCATA	GGTAGGGTCACTCTTGGCT
mRNF43	TCCGAAAGATCAGCAGAACAGA	GGACTGCATTAGCTTCCCTTC
mMito	ACTTCTAACTAAAAGAATTACAGC	TAGACGAGTTGATTCATAAAATTG
mNuc	CCTCAAGCATTCACCTCTTCTTTG	CCAAGGACCTGCTCGATGAC
mSdhb	TGGATCTGAATAAGTGCGGACC	GCCAGAGTATTGCCTCCGTT
mAtp5a1	CGGGACTGGTCTCCAAAAATG	CGTGTCAGCTCCCAGAATCC
mNdufb8	TGTTGCCGGGGTCATATCCTA	AGCATCGGGTAGTCGCCATA
mUqcrc2	AAAGTTGCCCCGAAGGTTAAA	GAGCATAGTTTTCCAGAGAAGCA
hRSPO1	CTGGCAAGGACTGGTGTTTGT	GGTTGATTGCCTCGACACCA
hUCP1	AGGTCCAAGGTGAATGCCC	TTACCACAGCGGTGATTGTTC
hPGC-1α	TCTGAGTCTGTATGGAGTGACAT	CCAAGTCGTTCACATCTAGTTCA
hDIO2	AGCAGACTACTGGTCTACTCAC	CACAGACTAATTTGCCTTGGGA
h-β-ACTIN	TGCGTGACATTAAGGAGAAG	GCTCGTAGCTCTTCTCCA
**shRNA**	**Sequencing**	
LV	TTCTCCGAACGTGTCACGT	
sh*Rspo1*	GCCACAACTTCTGCACCAAGT	

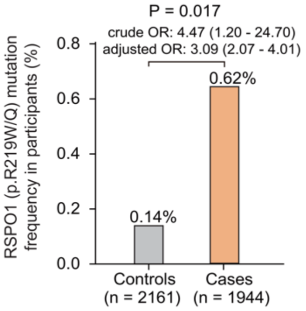

Figure S1. p.R219W/Q RSPO1 mutations were enriched in obese cases.

The frequency of RSPO1 mutations (p.R219W/Q) in lean subjects (controls, n = 2,161) versus young subjects with severe obesity (cases, n = 1,944).

Figure S2

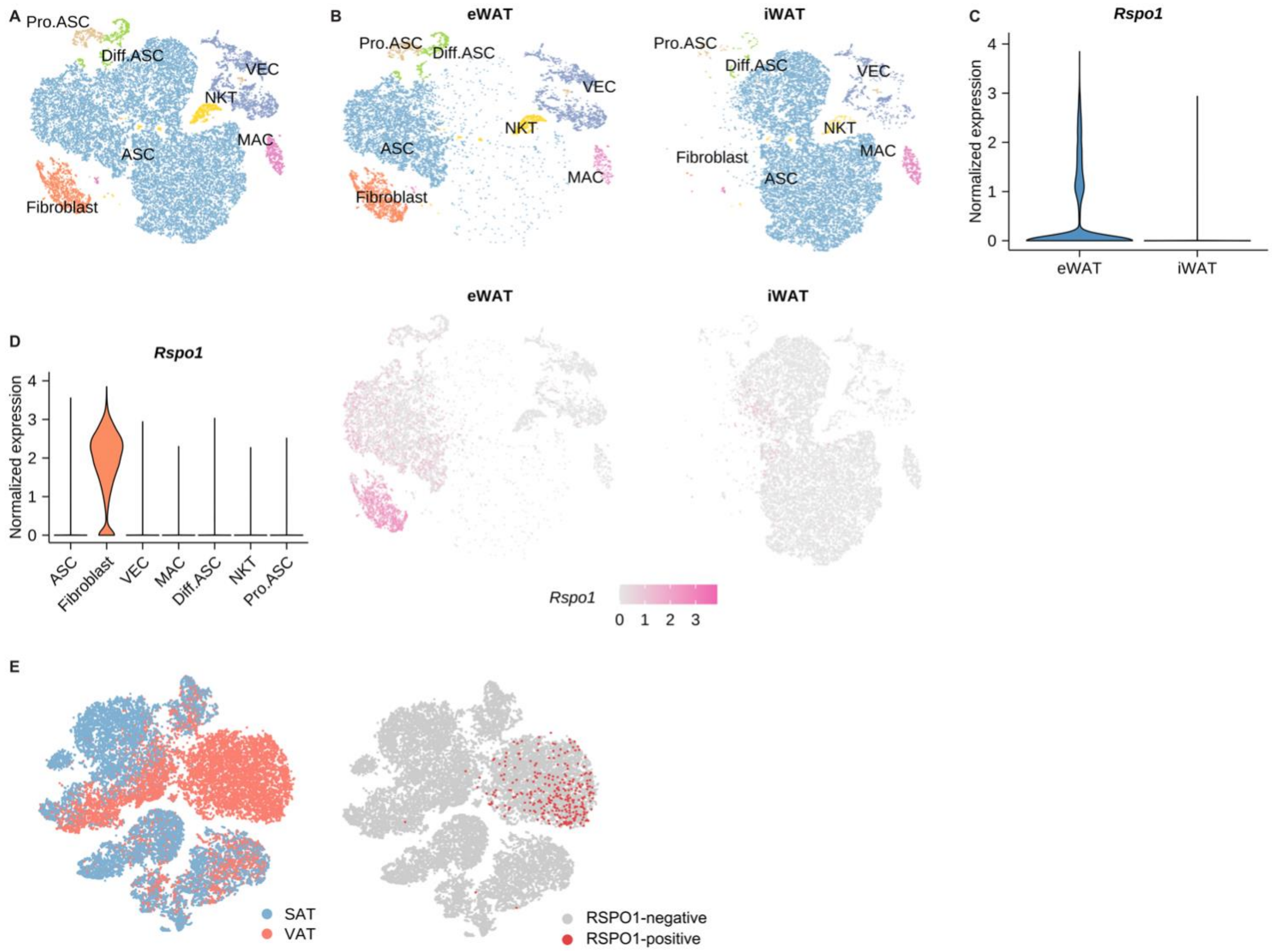

Figure S2. Rspo1 is highly expressed in fibroblasts, particularly in visceral fat.

A) Cell-type clusters of SVFs obtained from both eWAT and iWAT. K-means clustering was applied to the t-SNE reduced single-cell RNA sequencing data from a previous study (SRP145475). Cell types were identified by the enriched genes expression in each cluster. APCs, adipocyte progenitor cells; MAC, macrophages; VEC, vascular endothelial cells; NKT, natural killer T cells; Pro/Diff APCs, proliferating or differentiating adipocyte progenitor cells.

B) Upper panels: t-SNE plot of cell clusters from **Figure S2A** split into cells from eWAT and iWAT. Bottom panels: t-SNE plot highlighting the expression of *Rspo1* among different clusters. eWAT, epididymal white adipose tissue; iWAT, inguinal white adipose tissue.

C) Violin plots revealing the distinct expression distribution of *Rspo1* in SVFs derived from eWAT and iWAT.

D) Violin plots of *Rspo1* expression distribution among different clusters in SVF derived from eWAT, highly enriched in the fibroblast cluster.

E) t-SNE plot highlighting the expression of *RSPO1* in SVF cells isolated from human visceral adipose tissue (VAT) and subcutaneous adipose tissue (SAT).

Figure S3

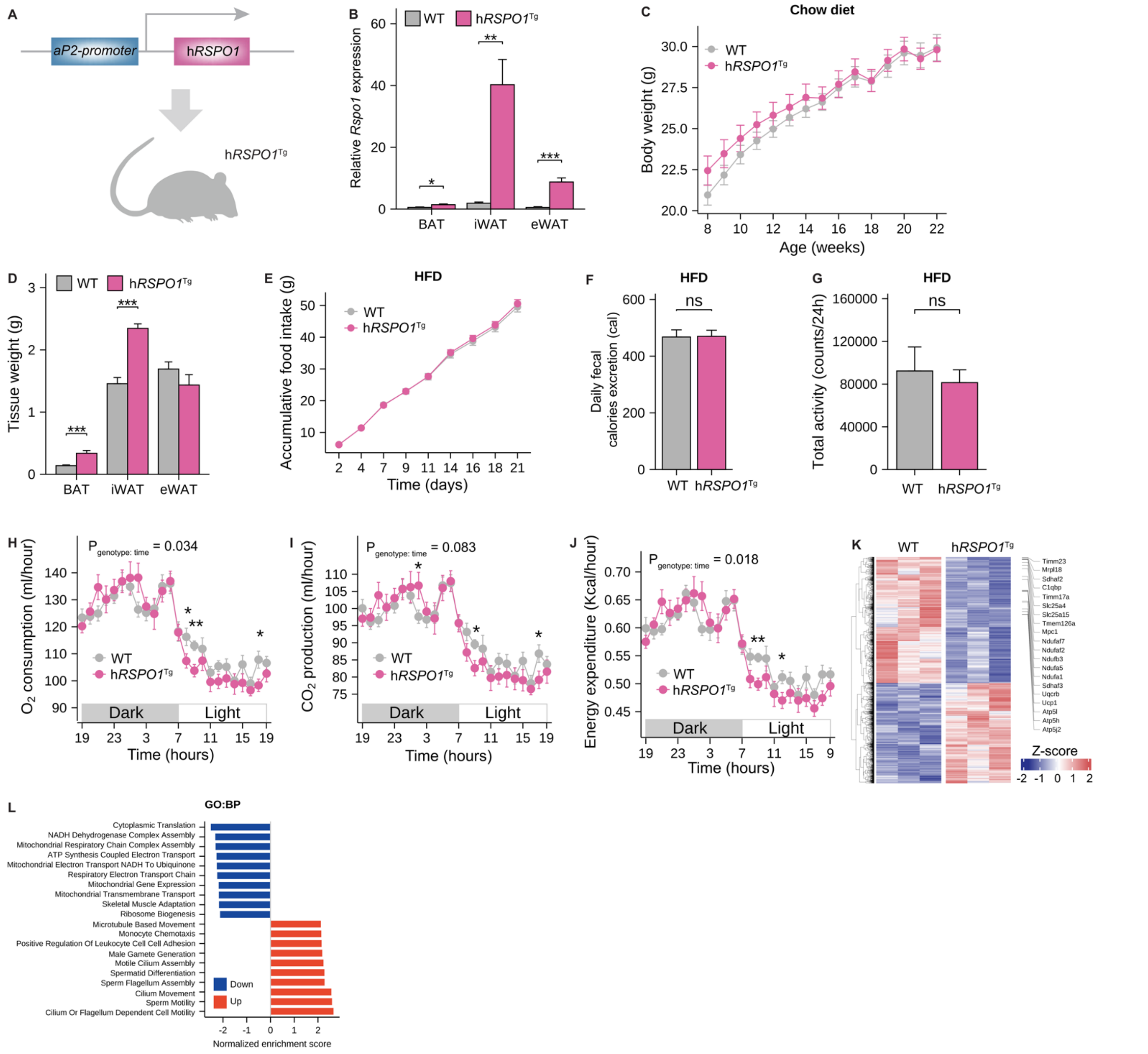

Figure S3. Human *RSPO1* overexpression promotes diet-induced adiposity *in vivo*.

A) Schematic of human *RSPO1* transgenic (h*RSPO1*^{Tg}) mice driven by mouse aP2 promoter.

B) Quantitative PCR analysis of h*RSPO1* expression in BAT, iWAT, and eWAT of h*RSPO1*^{Tg} and WT littermate mice (n = 4–5 per group).

C) Body weight curve of h*RSPO1*^{Tg} and WT mice fed with chow diet (n = 7–17 per group).

D) Fat tissue weight of h*RSPO1*^{Tg} and WT littermate mice fed HFD for 14 weeks (n = 8 per group).

E–G) Cumulative food intake (E) and daily fecal calorie loss (F) (n = 13–14 per group), and 24-hour physical activity (n = 8 per group) (G) of WT and h*RSPO1*^{Tg} mice fed with one-week HFD.

H–J) O_2 consumption (H), CO_2 production (I), and energy expenditure (J) of wild-type and h*RSPO1*^{Tg} mice fed with one-week HFD (n = 8 per group). The hourly measurements were assessed by two-way ANOVA model to evaluate the interaction between genotype and time, and pairwised t-test with Benjamini-Hochberg correction was used as post-hoc test to evaluate the differences between genotypes in each hour.

K) The heatmap of genes differentially expressed in BAT between h*RSPO1*^{Tg} and WT mice fed HFD (n = 3 per group). Representative genes related to mitochondrial biogenesis and function were labeled.

L) The most significant changed pathways revealed by GSEA analysis based on GO:BP database in BAT of h*RSPO1*^{Tg} mice.

Data are shown as the mean ± sem, and statistical significances between genotypes were assessed by unpaired Student's t test (B–G). FDR below 0.05 was considered as the criteria for evaluating differential expressed genes and pathways between genotypes (K–L). *$p < 0.05$; **$p < 0.01$; ***$p < 0.001$; ns, no significant ($p > 0.05$).

Figure S4

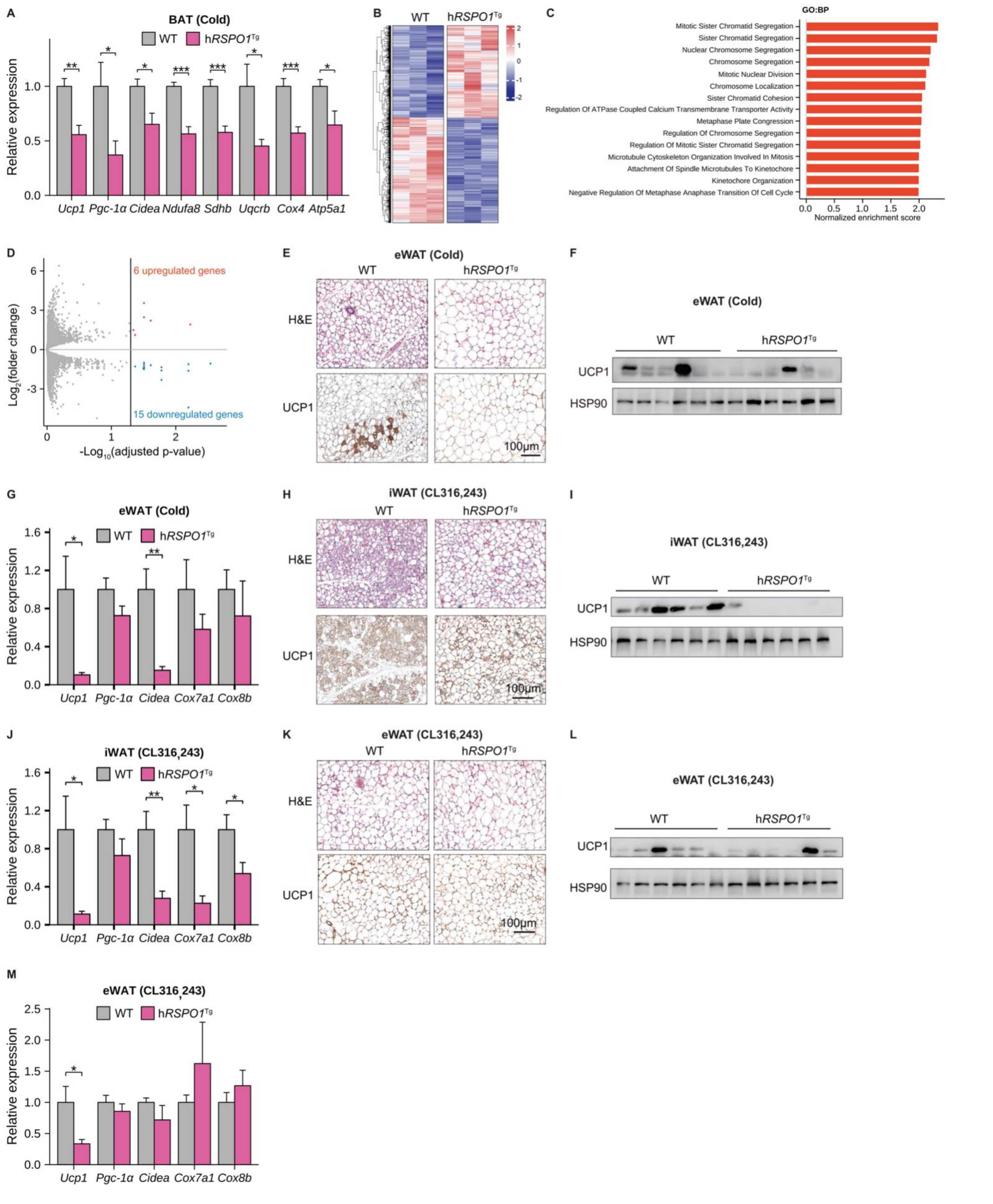

Figure S4. Human *RSPO1* overexpression represses thermogenic capacities of brown/beige fat *in vivo*.

A) The mRNA expression of thermogenic and mitochondrial genes in BAT of h*RSPO1*^{Tg} and WT mice exposed to cold (n = 7–8 per group).

B) The heatmap of genes differentially expressed in iWAT between h*RSPO1*^{Tg} and WT mice exposed to prolonged cold stimulation (n = 3 per group).

C) The top-upregulated pathways (FDR < 0.05) were revealed by GSEA analysis based on GO:BP database in iWAT of h*RSPO1*^{Tg} versus WT mice exposed to prolonged cold stimulation.

D) The volcano plot of genes differentially expressed in eWAT of h*RSPO1*^{Tg} versus WT mice exposed to prolonged cold stimulation.

E–G) Representative images of H&E staining and UCP1 immunohistochemical staining (E), Western blotting of UCP1 protein (F), and quantitative PCR analysis of mitochondria-related genes (G) in eWAT of WT and h*RSPO1*^{Tg} mice exposed to prolonged cold stimulation (n = 7–8 per group). Scale bar, 100 μm.

H–J) Representative images of H&E staining and UCP1 immunohistochemical staining (H), Western blotting of UCP1 protein (I), and quantitative PCR analysis of mitochondria-related genes (J) in iWAT of WT and h*RSPO1*^{Tg} mice under β3-AR agonist (CL316,243) injection for 7 days (n = 6–7 per group). Scale bar, 100 μm.

K–M) Representative images of H&E staining and UCP1 immunohistochemical staining (K), Western blotting of UCP1 protein (L), and quantitative PCR analysis of mitochondria-related genes (M) in eWAT of WT and h*RSPO1*^{Tg} mice under CL316,243 treatment for 7 days (n = 6–7 per group). Scale bar, 100 μm.

Data are shown as the mean ± sem, and statistical significance between genotypes was assessed by unpaired Student's t test (A, G, J, and M). FDR below 0.05 were considered as the criteria for evaluating differential expressed genes and pathway between genotypes (B–D). *p < 0.05; **p < 0.01; ***p < 0.001.

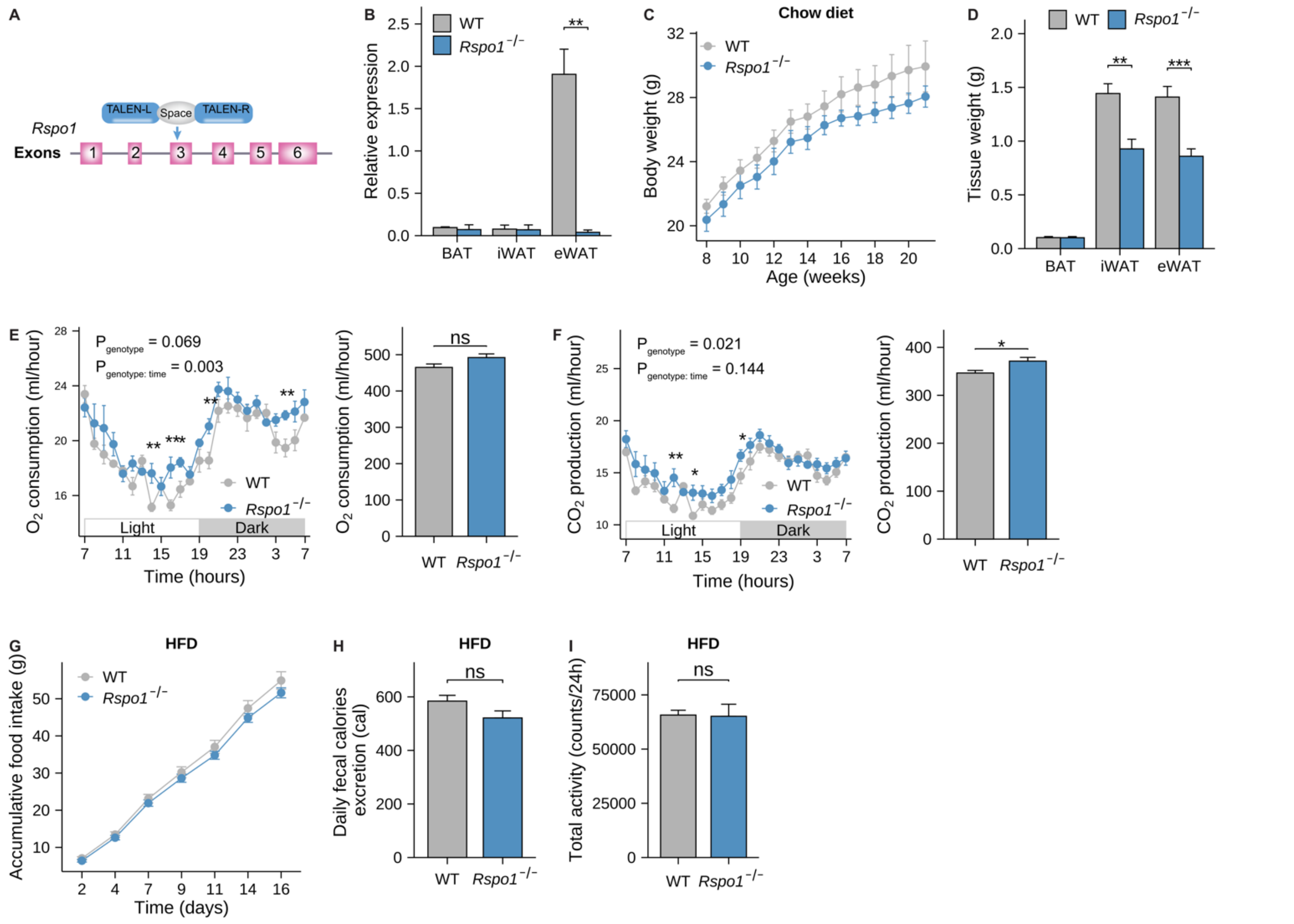

Figure S5. Ablation of endogenous *Rspo1* reduces adiposity and enhances energy expenditure.

A) Schematic of global *Rspo1* knockout (*Rspo1*$^{-/-}$) mice generated by TALEN method targeting the third exon of mouse *Rspo1* gene; red square frame indicates the six exons of mouse *Rspo1* gene.

B) Quantitative PCR analysis of Rspo1 expression in BAT, iWAT, and eWAT of *Rspo1*$^{-/-}$ and WT mice (n = 3 per group).

C) Body weight curve of *Rspo1*$^{-/-}$ and WT mice fed chow diet (n = 7–8 per group).

D) Weight of BAT, iWAT, and eWAT in *Rspo1*$^{-/-}$ and WT mice fed HFD (n = 7–8 per group)

E–F) Hourly (left) and total (right) O_2 consumption (E) and CO_2 production (F) over 24 hours of WT and *Rspo1*$^{-/-}$ mice fed with 1-week HFD (n = 8 per group). The hourly data was assessed by two-way ANOVA model to evaluate the interaction between genotype and time, and pairwised t-test with Benjamini-Hochberg correction was used as post-hoc test to examine the differences between genotypes in each hour.

G–I) Cumulative food intake (G) and daily fecal calorie loss (H) (n = 8–12 per group), and physical activity (n = 8 per group) (I) of *Rspo1*$^{-/-}$ and WT mice fed with HFD.

Data are shown as the mean ± sem, and statistical significance between genotypes was assessed by unpaired Student's t test (B–D, G–I). $^*p < 0.05$; $^{**}p < 0.01$; $^{***}p < 0.001$; ns, no significant ($p > 0.05$).

.

Figure S6

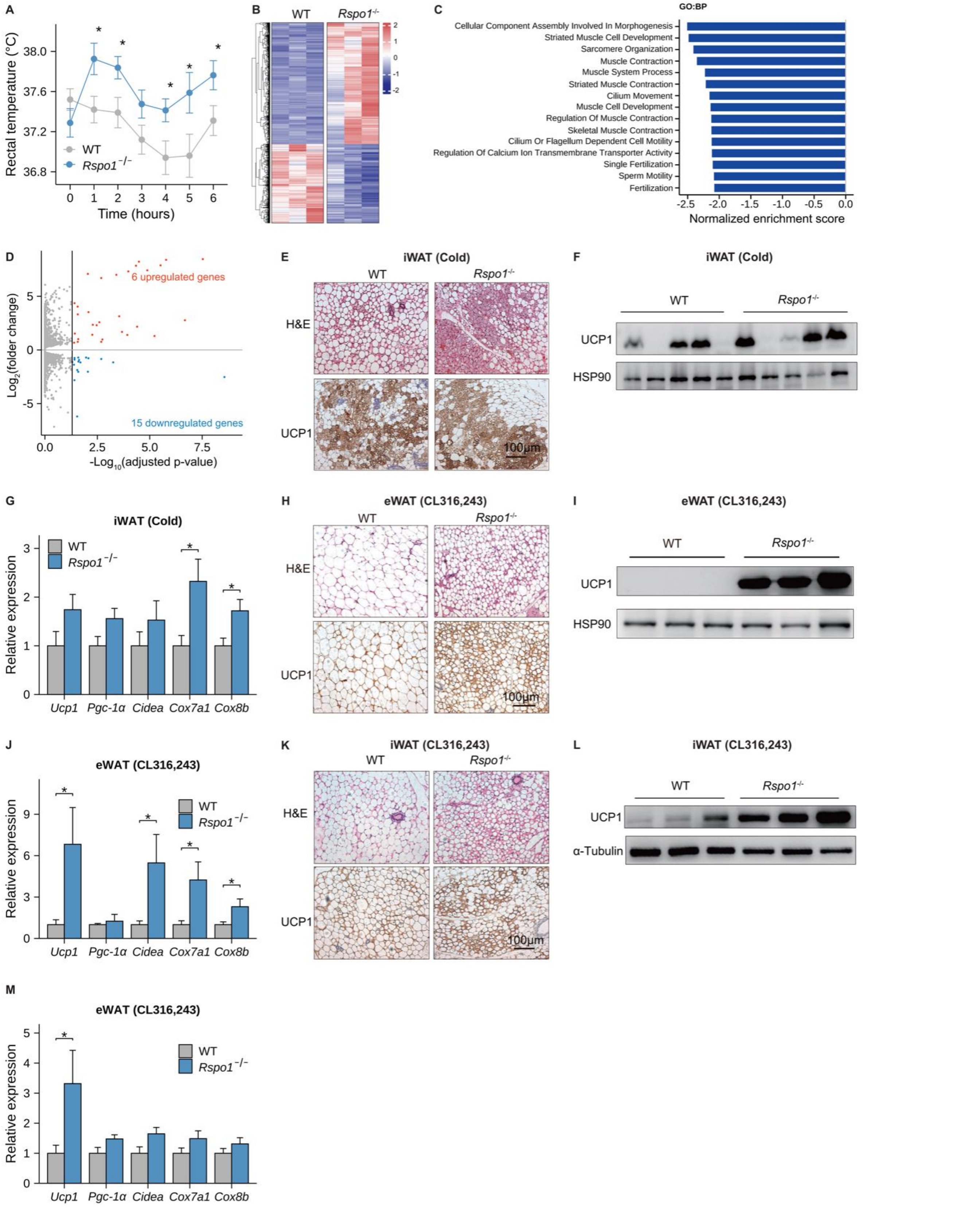

Figure S6. Ablation of *Rspo1* promotes browning program of white adipose tissue.

A) Rectal temperature changes of WT and *Rspo1*$^{-/-}$ mice in response to acute cold stimulation (4 °C) (n = 8–10 per group).

B) The heatmap of genes differentially expressed in eWAT between *Rspo1*$^{-/-}$ versus WT mice under prolonged cold stimulation (4 °C) for 10 days (n = 3 per group).

C) The top-downregulated pathways (FDR < 0.05) revealed by GSEA based on GO:BP database in eWAT of *Rspo1*$^{-/-}$ versus WT mice under prolonged cold stimulation.

D) The volcano plot of genes differentially expressed in iWAT of *Rspo1*$^{-/-}$ versus WT mice under prolonged cold stimulation.

E–G) Representative images of H&E staining and UCP1 immunohistochemical staining (E), Western blotting of UCP1 protein (F), and quantitative PCR analysis of mitochondria-related genes (G) in iWAT of WT and *Rspo1*$^{-/-}$ mice under prolonged cold stimulation (n = 7–8 per group). Scale bar, 100 μm.

H–J) Representative images of H&E staining and UCP1 immunohistochemical staining (H), Western blotting of UCP1 protein (I), and quantitative PCR analysis of mitochondria-related genes (J) in the eWAT of WT and *Rspo1*$^{-/-}$ mice under CL316,243 treatment for 7 days (n = 4–5 per group). Scale bar, 100 μm.

K–M) Representative images of H&E staining and UCP1 immunohistochemical staining (K), Western blotting of UCP1 protein (L), and quantitative PCR analysis of mitochondria-related genes (M) in the iWAT of WT and *Rspo1*$^{-/-}$ mice under CL316,243 treatment for 7 days (n = 4–5 per group). Scale bar, 100 μm.

Data are shown as the mean ± sem, and statistical significance between genotypes was assessed by unpaired Student's t test (A, G, J, and M). FDR below 0.05 was considered as the criteria for evaluating differential expressed genes and pathways between genotypes (B–D). *p < 0.05; **p < 0.01; ***p < 0.001.

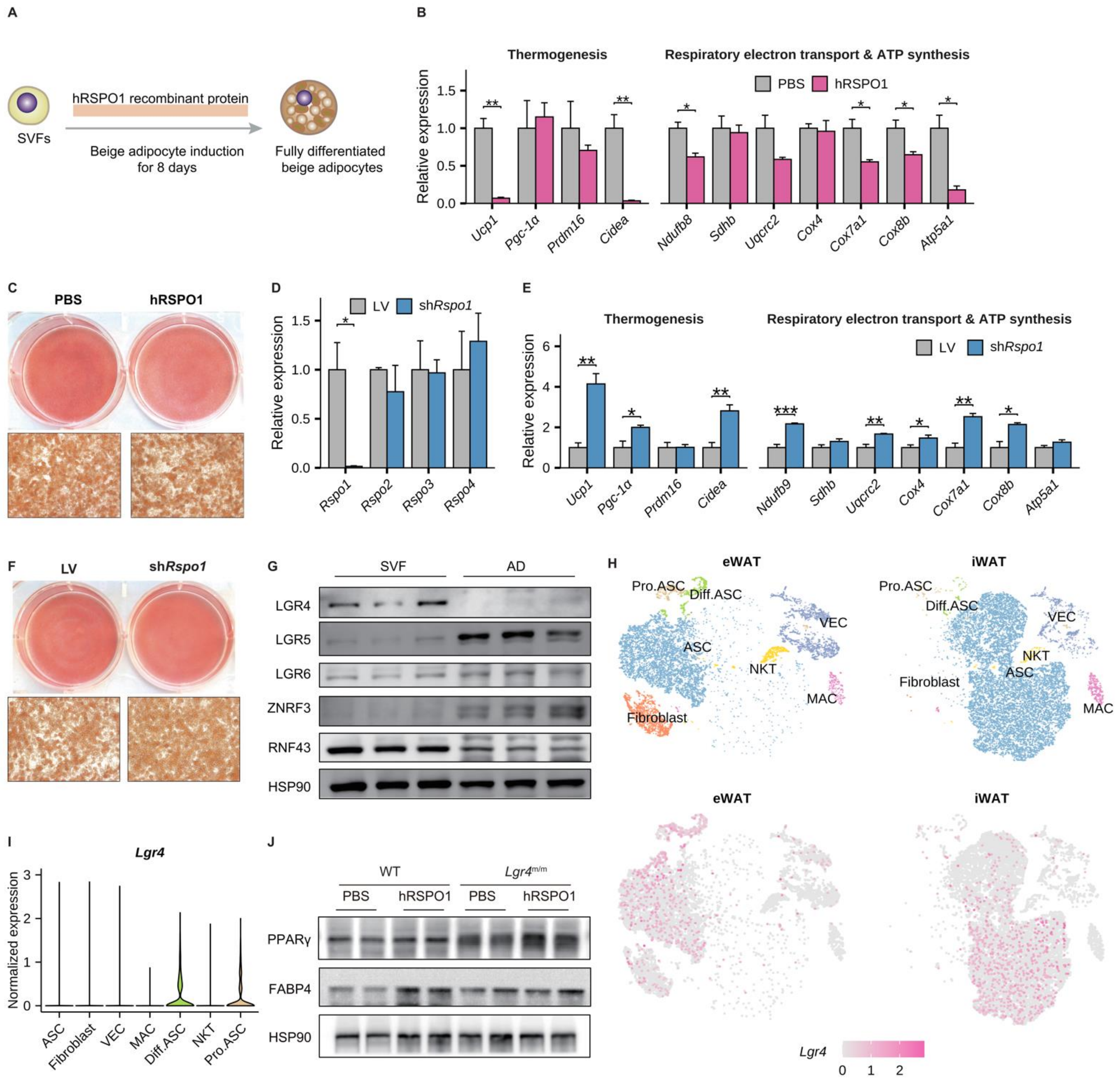

Figure S7. RSPO1 inhibits thermogenic gene expression in induced beige adipocytes.

A) Schematic of experimental design for the induction of beige adipocytes derived from SVF cells of iWAT.

B and C) Quantitative PCR analysis of genes of thermogenesis and mitochondrial respiratory complexes (n = 3 per group) (B) and Oil O Red staining (C) in fully differentiated beige adipocytes in response to hRSPO1 treatment.

D) Quantitative PCR analysis of *Rspo1–4* genes in SVF cells infected with the mouse *Rspo1* shRNA-lentivirus (sh*Rspo1*) or lentivirus vector (LV) (n = 3 per group).

E and F) Quantitative PCR analysis of genes of thermogenesis and mitochondrial respiratory complexes (n = 4 per group) (E) and Oil Red O staining (F) of fully differentiated beige adipocytes derived from SVF cells infected with sh*Rspo1* or LV before induction.

G) Western blotting of membrane receptor candidates of RSPO1, including LGR4/5/6 and ZNRF3/RNF43 E3 ligases, in SVF fractions and mature adipocytes of eWAT.

H) The t-SNE plot of cell-type clusters from eWAT and iWAT, respectively (upper panels), and the t-SNE plot highlighting the expression of *Lgr4* among different clusters (bottom panels). Cell-type clusters were referred to **Figure S2A**.

I) The violin plot of *Lgr4* expression distribution among different clusters in SVF derived from eWAT.

J) Western blotting analysis of PPARγ and FABP4 proteins in fully differentiated beige adipocytes derived from WT and *Lgr4*$^{m/m}$ mice in response to hRSPO1 or PBS treatment, respectively.

hRSPO1, recombinant human RSPO1 protein. Data are shown as the mean ± sem, and statistical significance between groups was assessed by unpaired Student's t test (B, D, and E). *p < 0.05; **p < 0.01; ***p < 0.001.

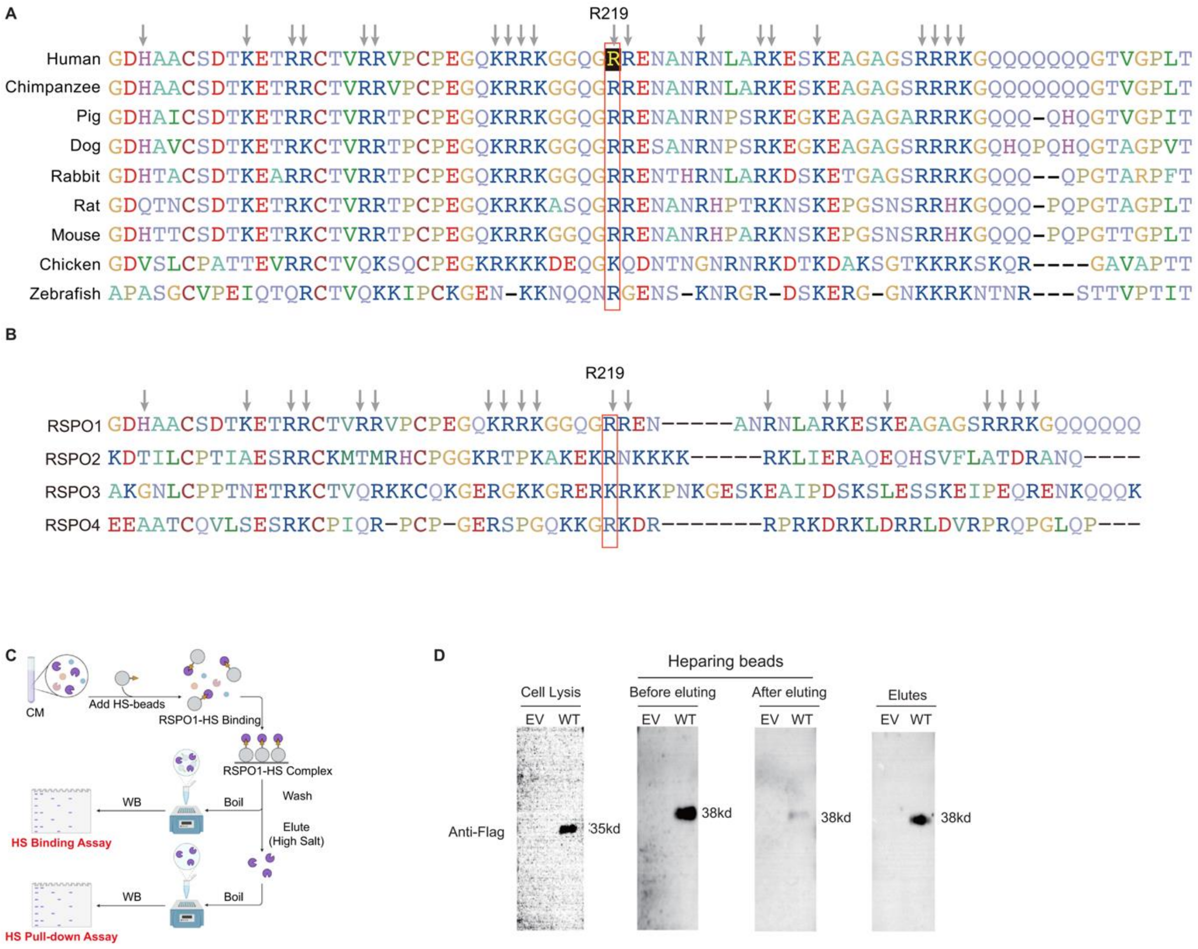

Figure S8. The R219 residue contained in the conserved C-terminal region of human RSPO1 contributes to its interaction with HSPG.

A and B) The consensus sequence of the positively charged residues (nearby R219) in the C-terminal region of human RSPO1 protein is highly conserved across various species (A) but relatively mildly conserved among four R-spondin members (B).

C) Schematic protocol of RSPO1–heparan sulfate (HS) binding and pull-down assay, graphics were created with BioRender.com.

D) Western blotting of flagged RSPO1 protein in the cell lysates of HEK293T cells transfected with wild-type RSPO1 plasmids (the left panel), flagged RSPO1–HS complex in the cell lysates incubated with HS beads (before and after 1.2M NaCl elusion, the middle two panels), and flagged RSPO1–HS complex in the elutes (the right panel).

Figure S9

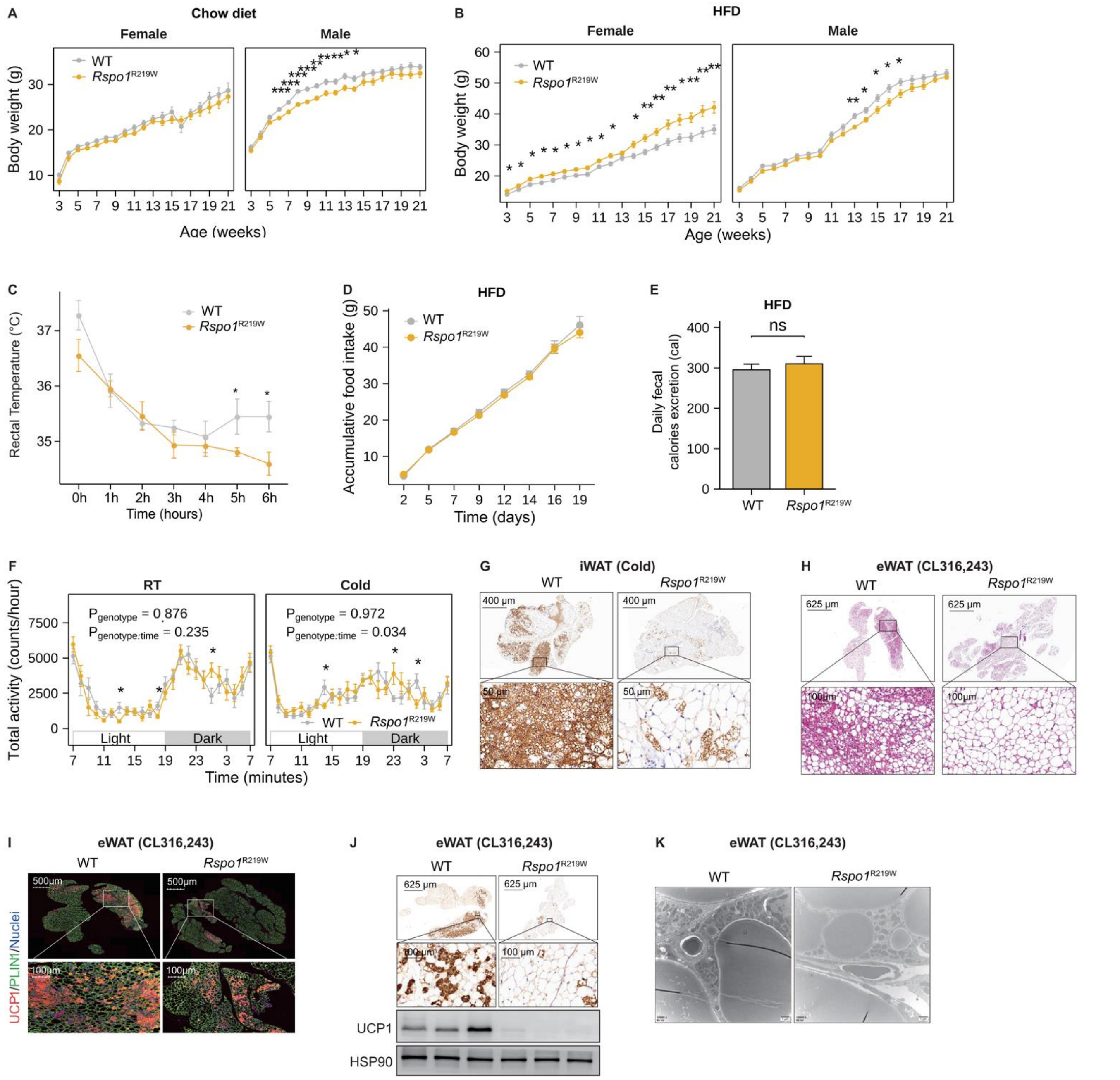

Figure S9. Rspo1 p.R219W mutation inhibits cold- and β3AR agonist-induced thermogenesis.

A and B) Body weight curve of homozygous *Rspo1*^{R219W} and WT littermate mice fed chow diet (n = 10 per group for females, n = 7–8 per group for males) (A) or HFD (n = 9–11 per group for females, n = 12 per group for males) (B), respectively.

C) Rectal temperature changes of WT and *Rspo1*^{R219W} mice in response to acute cold stimulation (4 C°) (n = 6–8 per group).

D–F) Cumulative food intake (D) and daily fecal calorie loss (E) (n =14 per group), and physical activities (n = 12 per group) (F) of female WT and *Rspo1*^{R219W} mice fed HFD. The repeated measurements of physical

activities were assessed by two-way ANOVA model to evaluate the interaction between genotype and time, and pairwised t-test with Benjamini-Hochberg correction was used as post-hoc test for evaluating the differences between genotypes in each hour.

G) Representative images of UCP1 immunohistochemical staining in iWAT of female WT and *Rspo1*^{R219W} mice exposed to cold stimulus (4 C°) for 10 days (n = 3 per group). Scale bars were indicated in the panels.

H–K) Representative images of H&E staining (H), UCP1 immunofluorescent staining (I), the immunohistochemical staining and Western blotting of UCP1 protein (J), and electron microscopic images (K) in eWAT of female WT and *Rspo1*^{R219W} mice under CL316,243 treatment (n = 3 per group). UCP1 (red) and Perilipin protein (green) were used to mark beige adipocytes and lipid droplets, respectively. Scale bars were indicated in the panels.

Data are shown as the mean ± sem, and statistical significance between groups was assessed by unpaired Student’s t-test (A–E). *p < 0.05; **p < 0.01; ***p < 0.001; ns, no significant (p >0.05).

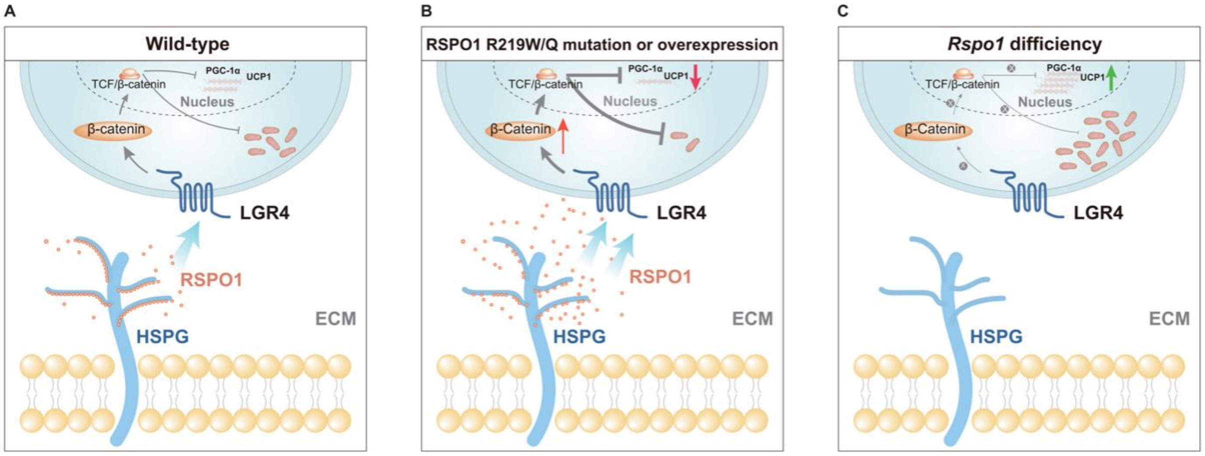

Figure S10. Schematic models of RSPO1 suppressing the browning program of targeted preadipocytes surrounding RSPO1-secreting cells in a paracrine way. WNT-related factors are regarded to act as morphogen molecules, which form concentration gradients as they diffuse from the site of their synthesis,[1] while p.R219W/Q mutations in human RSPO1 protein disrupt the hemostasis of local concentration gradients and consequent diffusion distance. The mutations provide the first clinical evidence of the etiology of disturbed RSPO1/WNTs-ECM electrostatic interaction in the development of human diseases.

A) In physiological status, a small proportion of RSPO1 protein from RSPO1-secreting cells can escape from the electrostatic interaction with the components of ECM (like HSPG), especially in visceral fat where RSPO1 is robustly expressed, and reaches neighboring adipocyte progenitors positive for LGR4 receptor. The RSPO1 binding to LGR4 amplifies canonical Wnt/β-catenin signaling that thereby represses the mitochondrial biogenesis and thermogenic capacity.

B) In pathological conditions, p.R219W/Q mutations disrupt the electrostatic interaction between RSPO1 and HSPG, resulting in long-distance diffusion and more arrivals of RSPO1 protein to LGR4-positive progenitor cells, and consequently enhance their inhibition on mitochondrial biogenesis and thermogenesis during browning program, contributing to the development of adiposity. The Rspo1 p.R219W mutant mice are applied to resemble the pathophysiological changes of obese cases carrying RSPO1 p.R219W mutation, and faithfully recapitulate the clinical features of adiposity. Meanwhile, mice overexpressed human RSPO1 (h*RSPO1*^{Tg} mice) also well mimic the effect of excessive RSPO1 expression in the fat tissue of obese cases.

C) In an intervention model, *Rspo1* deletion or neutralization disinhibits its effects on the mitochondrial abundance and thermogenic capacity of brown/beige adipocytes, and consequently reduces and eventually combats adiposity.

Reference

[1] a) H. F. Farin, I. Jordens, M. H. Mosa, O. Basak, J. Korving, D. V. Tauriello, K. de Punder, S. Angers, P. J. Peters, M. M. Maurice, H. Clevers, *Nature* **2016**, *530* (7590), 340; b) G. Morata, G. Struhl, *Nature* **2014**, *505*, 162.
